# Supplementary material for: A Humanized Yeast Phenomic Model of Deoxycytidine Kinase to Predict Genetic Buffering of Nucleoside Analog Cytotoxicity
Source: Genes (Basel). 2019 Sep 30;10(10):770. doi: 10.3390/genes10100770 (PMC6826991; doi:10.3390/genes10100770)
Supplement: Supplementary file 1 [file genes-10-00770-s001.zip › Additional_Files/Additional_File7_GOTermHeatmaps.zip!/Additional_File7_GOTermHeatmaps/nucleobase-containing_compound_transport.pdf]

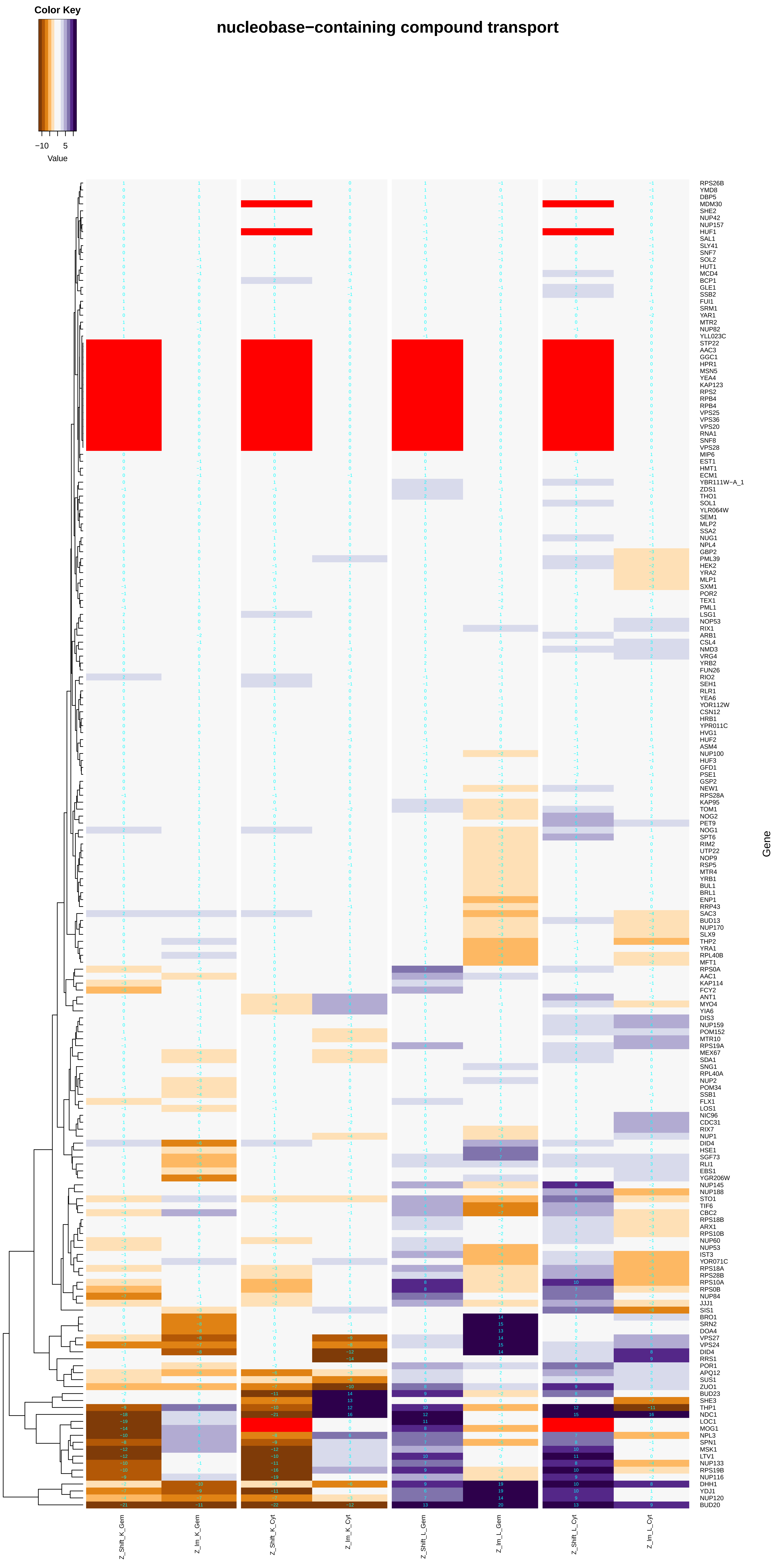
Color Key
−10
5
Value
nucleobase−containing compound transport
RPS26B
YMD8
DBP5
MDM30
SHE2
NUP42
NUP157
HUF1
SAL1
SLY41
SNF7
SOL2
HUT1
MCD4
BCP1
GLE1
SSB2
FUI1
SRM1
YAR1
MTR2
NUP82
YLL023C
STP22
AAC3
GGC1
HPR1
MSN5
YEA4
KAP123
RPS2
RPB4
RPB4
VPS25
VPS36
VPS20
RNA1
SNF8
VPS28
MIP6
EST1
HMT1
ECM1
YBR111W−A_1
ZDS1
THO1
SOL1
YLR064W
SEM1
MLP2
SSA2
NUG1
NPL4
GBP2
PML39
HEK2
YRA2
MLP1
SXM1
POR2
TEX1
PML1
LSG1
NOP53
RIX1
ARB1
CSL4
NMD3
VRG4
YRB2
FUN26
RIO2
SEH1
RLR1
YEA6
YOR112W
CSN12
HRB1
YPR011C
HVG1
HUF2
ASM4
NUP100
HUF3
GFD1
PSE1
GSP2
NEW1
RPS28A
KAP95
TOM1
NOG2
PET9
NOG1
SPT6
RIM2
UTP22
NOP9
RSP5
MTR4
YRB1
BUL1
BRL1
ENP1
RRP43
SAC3
BUD13
NUP170
SLX9
THP2
YRA1
RPL40B
MFT1
RPS0A
AAC1
KAP114
FCY2
ANT1
MYO4
YIA6
DIS3
NUP159
POM152
MTR10
RPS19A
MEX67
SDA1
SNG1
RPL40A
NUP2
POM34
SSB1
FLX1
LOS1
NIC96
CDC31
RIX7
NUP1
DID4
HSE1
SGF73
RLI1
EBS1
YGR206W
NUP145
NUP188
STO1
TIF6
CBC2
RPS18B
ARX1
RPS10B
NUP60
NUP53
IST3
YOR071C
RPS18A
RPS28B
RPS10A
RPS0B
NUP84
JJJ1
SIS1
BRO1
SRN2
DOA4
VPS27
VPS24
DID4
RRS1
POR1
APQ12
SUS1
ZUO1
BUD23
SHE3
THP1
NDC1
LOC1
MOG1
NPL3
SPN1
MSK1
LTV1
NUP133
RPS19B
NUP116
DHH1
YDJ1
NUP120
BUD20
Gene
Z_Shift_K_Gem
Z_lm_K_Gem
Z_Shift_K_Cyt
Z_lm_K_Cyt
Z_Shift_L_Gem
Z_lm_L_Gem
Z_Shift_L_Cyt
Z_lm_L_Cyt

nucleotide transport

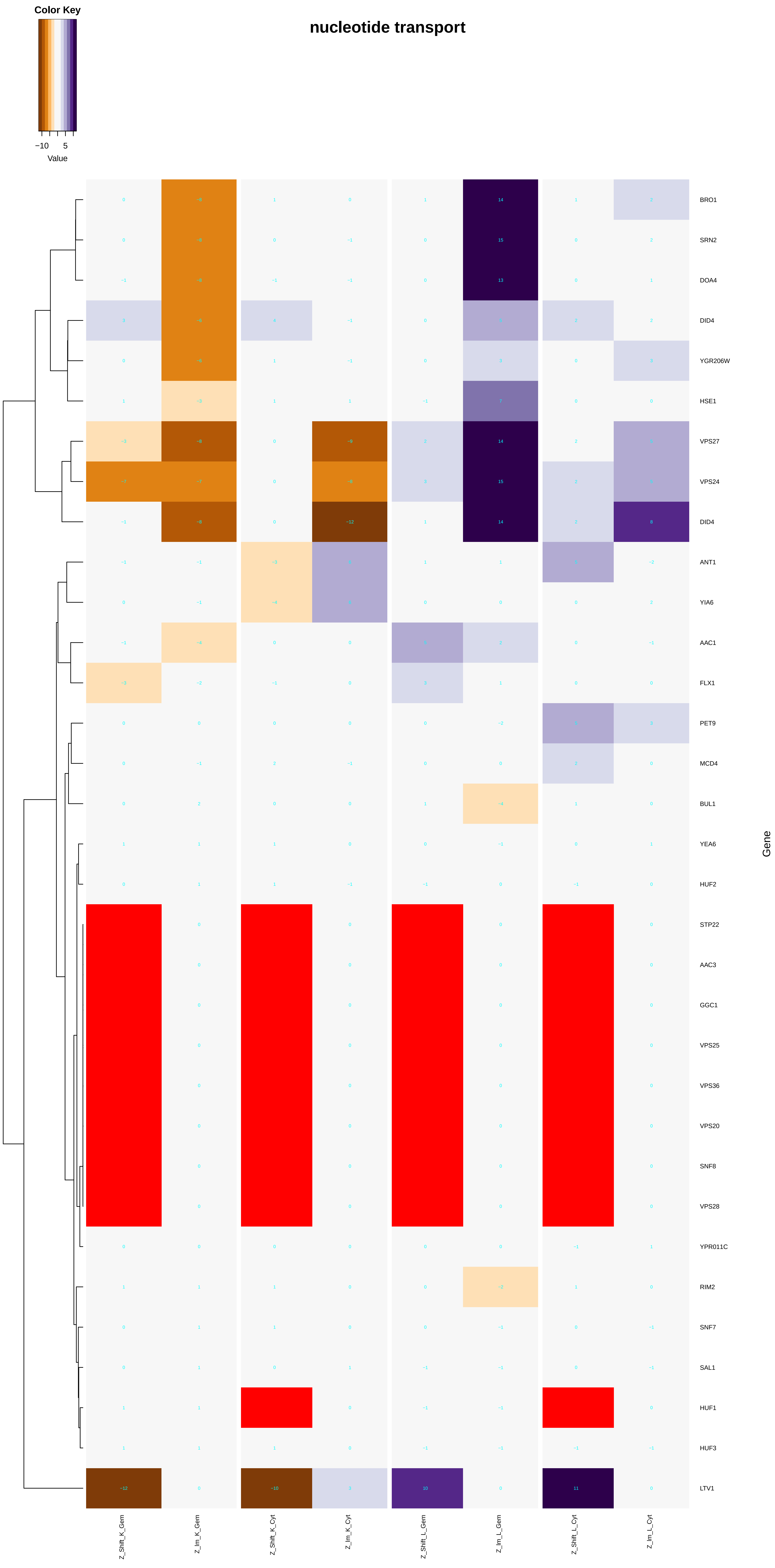

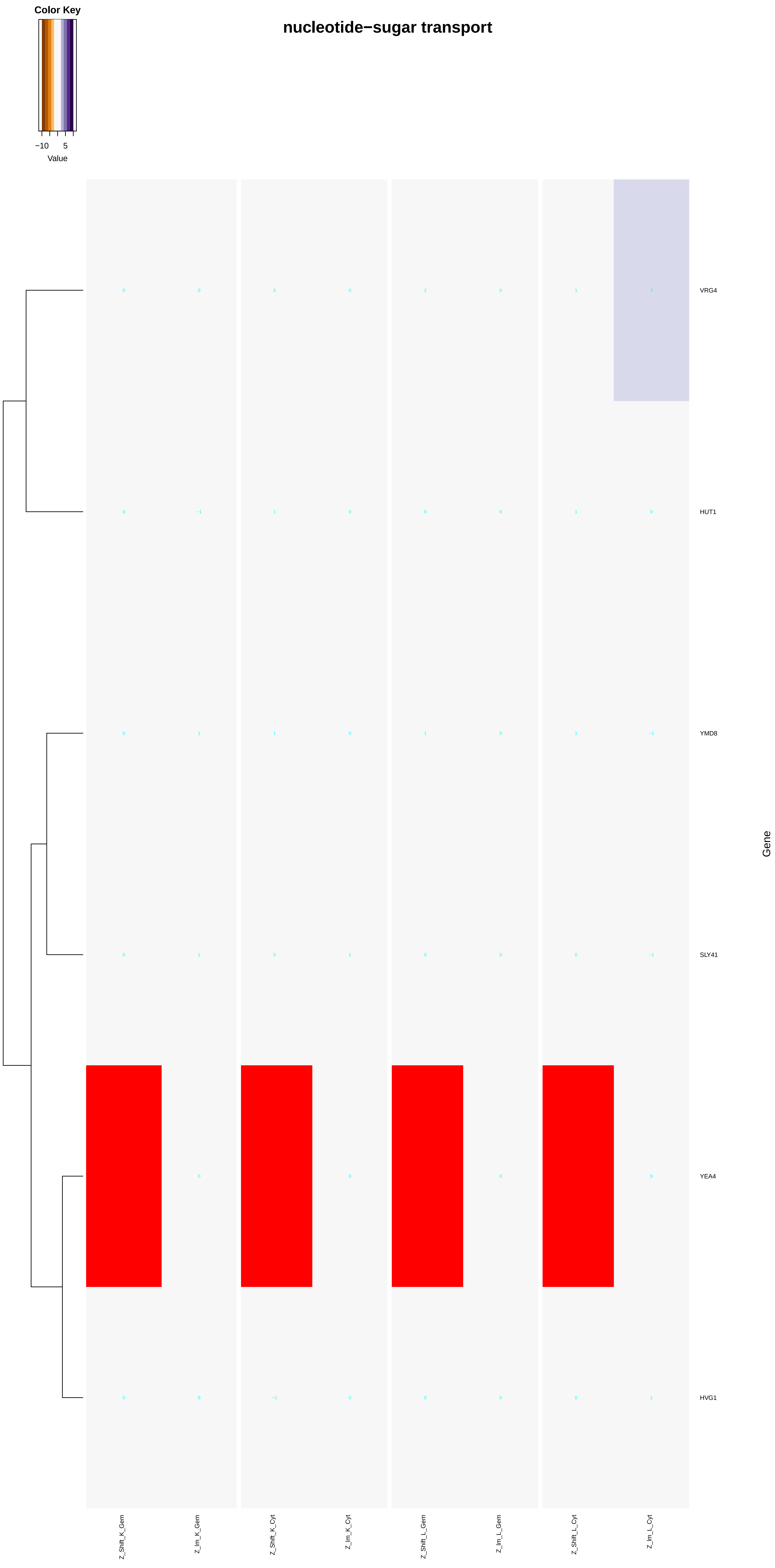
Color Key
−10
5
Value
nucleotide−sugar transport
VRG4
HUT1
YMD8
SLY41
YEA4
HVG1
Gene
Z_Shift_K_Gem
Z_Im_K_Gem
Z_Shift_K_Cyt
Z_Im_K_Cyt
Z_Shift_L_Gem
Z_Im_L_Gem
Z_Shift_L_Cyt
Z_Im_L_Cyt

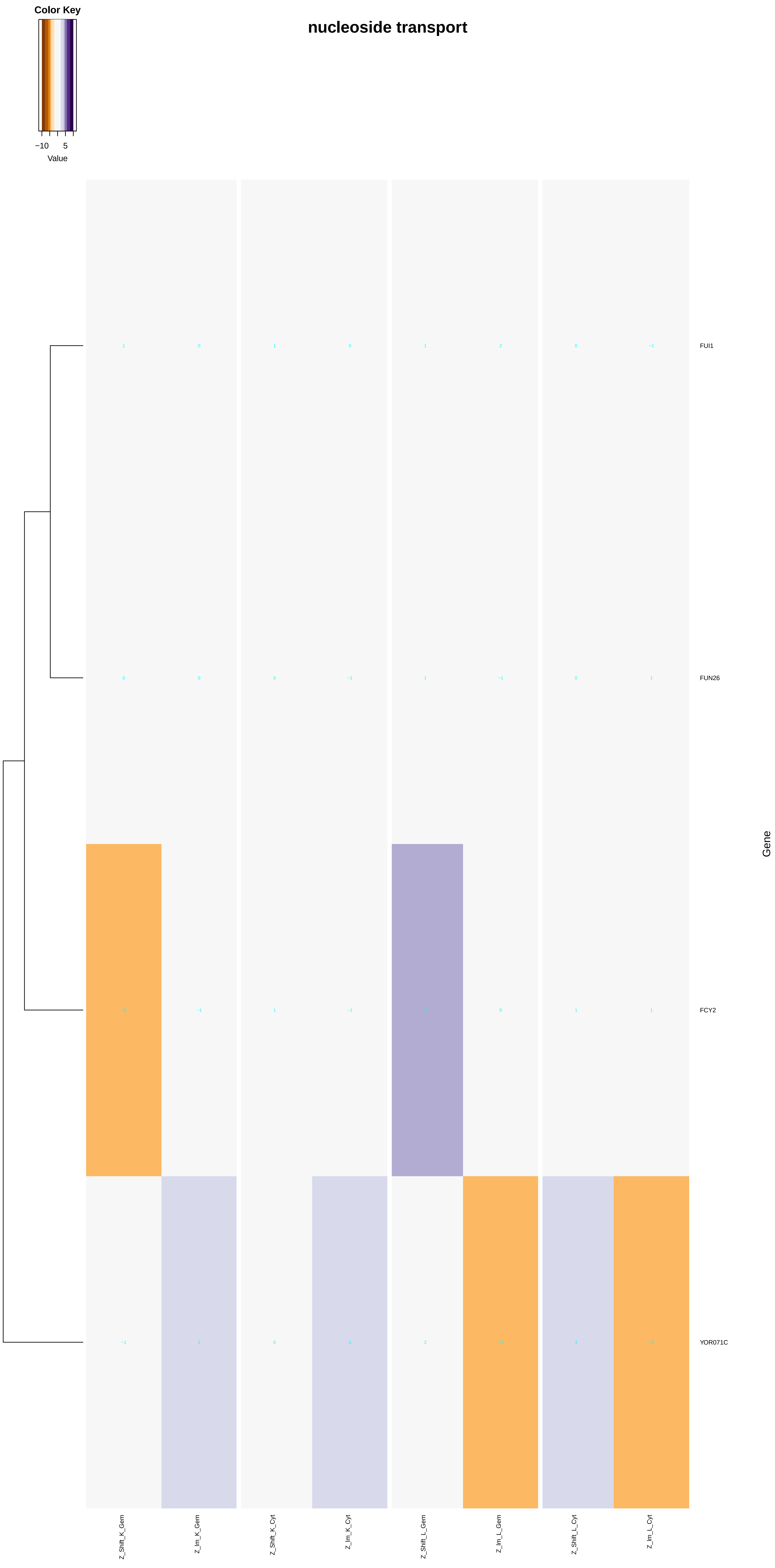

nucleoside transport
Color Key
−10
5
Value
FUI1
FUN26
FCY2
YOR071C
Gene
Z_Shift_K_Gem
Z_lm_K_Gem
Z_Shift_K_Cyt
Z_lm_K_Cyt
Z_Shift_L_Gem
Z_lm_L_Gem
Z_Shift_L_Cyt
Z_lm_L_Cyt

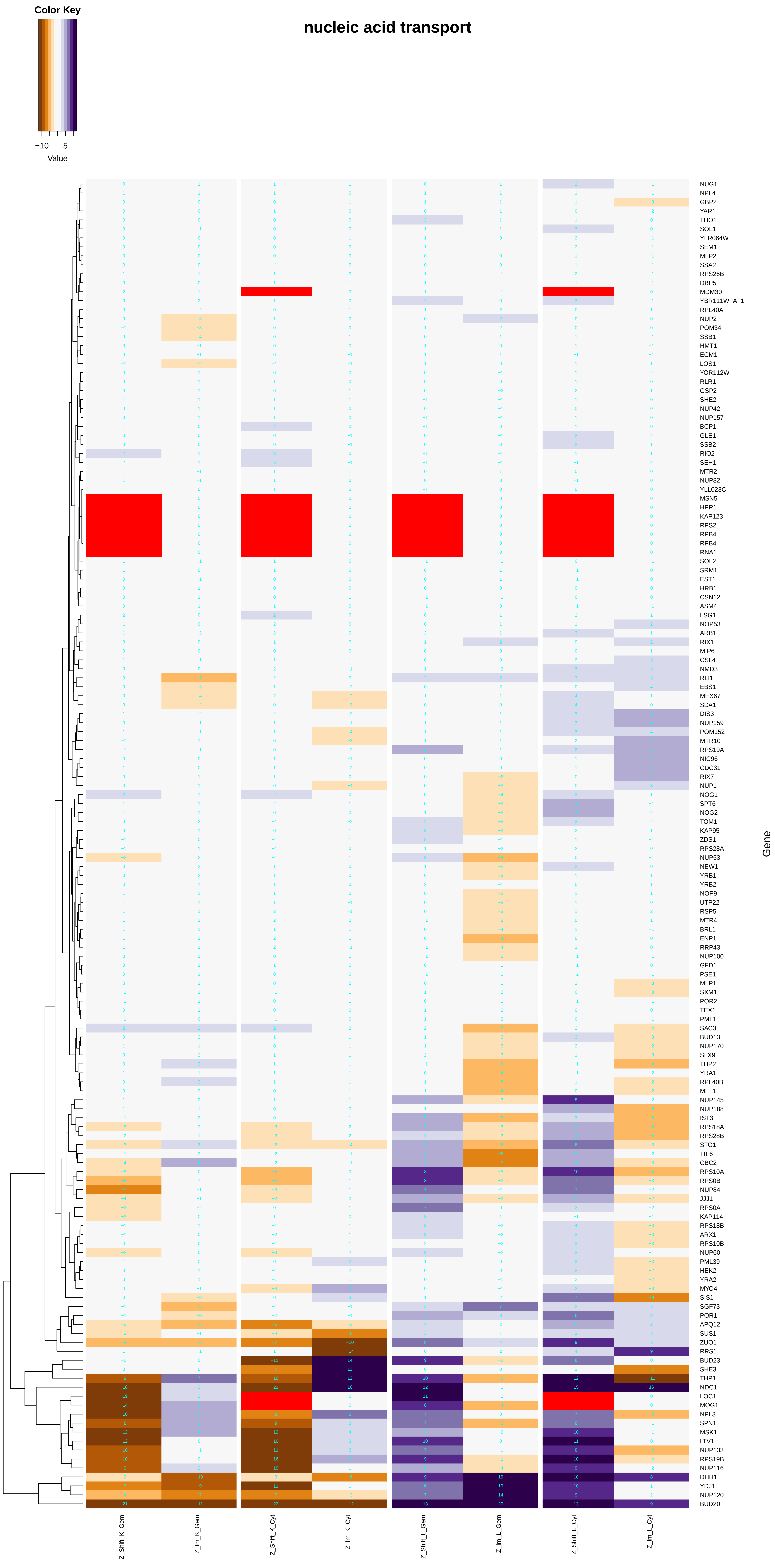
nucleic acid transport
Color Key
−10
5
Value
NUG1
NPL4
GBP2
YAR1
THO1
SOL1
YLR064W
SEM1
MLP2
SSA2
RPS26B
DBP5
MDM30
YBR111W−A_1
RPL40A
NUP2
POM34
SSB1
HMT1
ECM1
LOS1
YOR112W
RLR1
GSP2
SHE2
NUP42
NUP157
BCP1
GLE1
SSB2
RIO2
SEH1
MTR2
NUP82
YLL023C
MSN5
HPR1
KAP123
RPS2
RPB4
RPB4
RNA1
SOL2
SRM1
EST1
HRB1
CSN12
ASM4
LSG1
NOP53
ARB1
RIX1
MIP6
CSL4
NMD3
RLI1
EBS1
MEX67
SDA1
DIS3
NUP159
POM152
MTR10
RPS19A
NIC96
CDC31
RIX7
NUP1
NOG1
SPT6
NOG2
TOM1
KAP95
ZDS1
RPS28A
NUP53
NEW1
YRB1
YRB2
NOP9
UTP22
RSP5
MTR4
BRL1
ENP1
RRP43
NUP100
GFD1
PSE1
MLP1
SXM1
POR2
TEX1
PML1
SAC3
BUD13
NUP170
SLX9
THP2
YRA1
RPL40B
MFT1
NUP145
NUP188
IST3
RPS18A
RPS28B
STO1
TIF6
CBC2
RPS10A
RPS0B
NUP84
JJJ1
RPS0A
KAP114
RPS18B
ARX1
RPS10B
NUP60
PML39
HEK2
YRA2
MYO4
SIS1
SGF73
POR1
APQ12
SUS1
ZUO1
RRS1
BUD23
SHE3
THP1
NDC1
LOC1
MOG1
NPL3
SPN1
MSK1
LTV1
NUP133
RPS19B
NUP116
DHH1
YDJ1
NUP120
BUD20
Gene
Z_Shift_K_Gem
Z_Im_K_Gem
Z_Shift_K_Cyt
Z_Im_K_Cyt
Z_Shift_L_Gem
Z_Im_L_Gem
Z_Shift_L_Cyt
Z_Im_L_Cyt

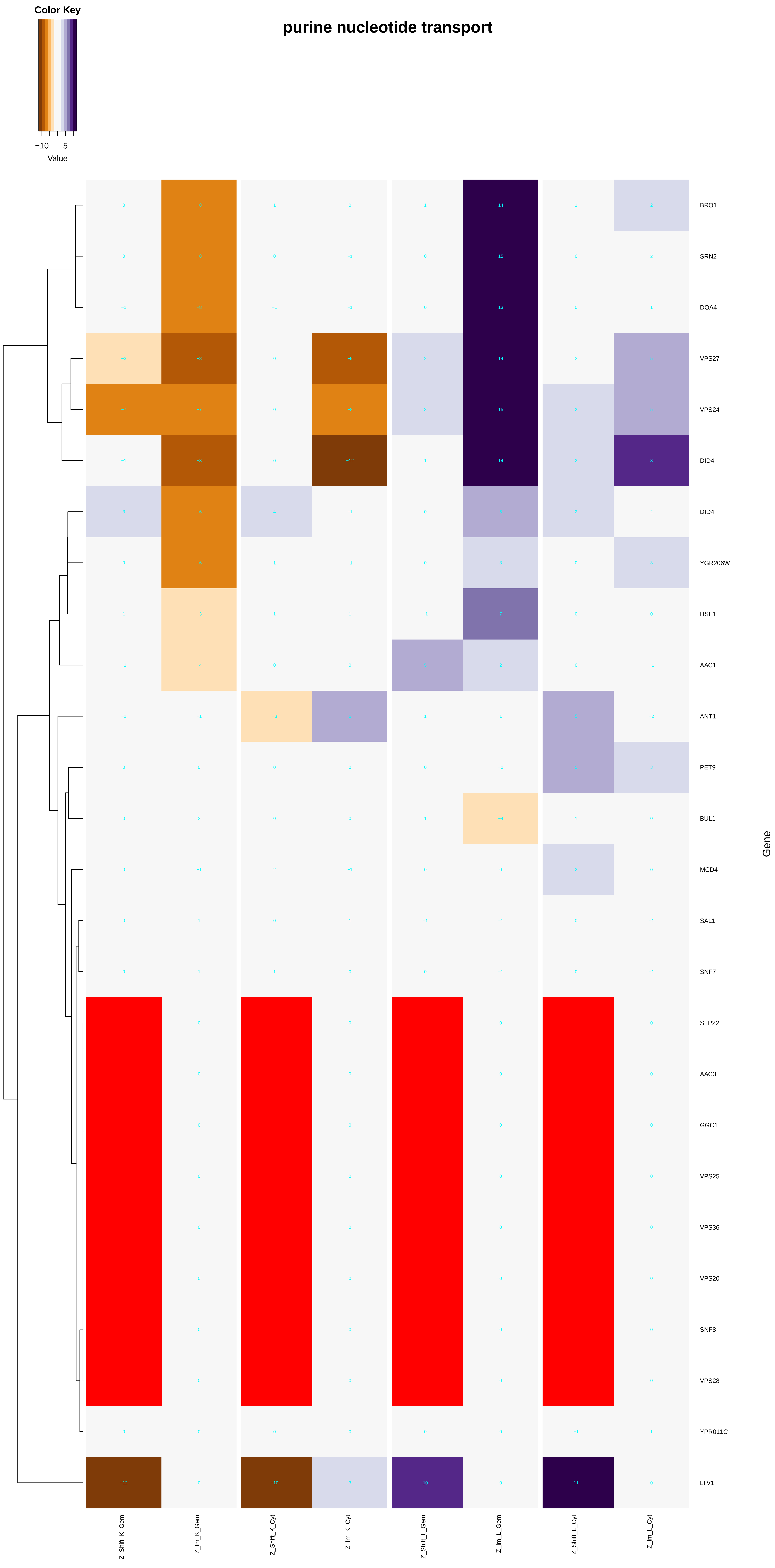

Color Key
−10
5
Value
purine nucleotide transport
BRO1
SRN2
DOA4
VPS27
VPS24
DID4
DID4
YGR206W
HSE1
AAC1
ANT1
PET9
BUL1
MCD4
SAL1
SNF7
STP22
AAC3
GGC1
VPS25
VPS36
VPS20
SNF8
VPS28
YPR011C
LTV1
Gene
Z_Shift_K_Gem
Z_Im_K_Gem
Z_Shift_K_Cyt
Z_Im_K_Cyt
Z_Shift_L_Gem
Z_Im_L_Gem
Z_Shift_L_Cyt
Z_Im_L_Cyt

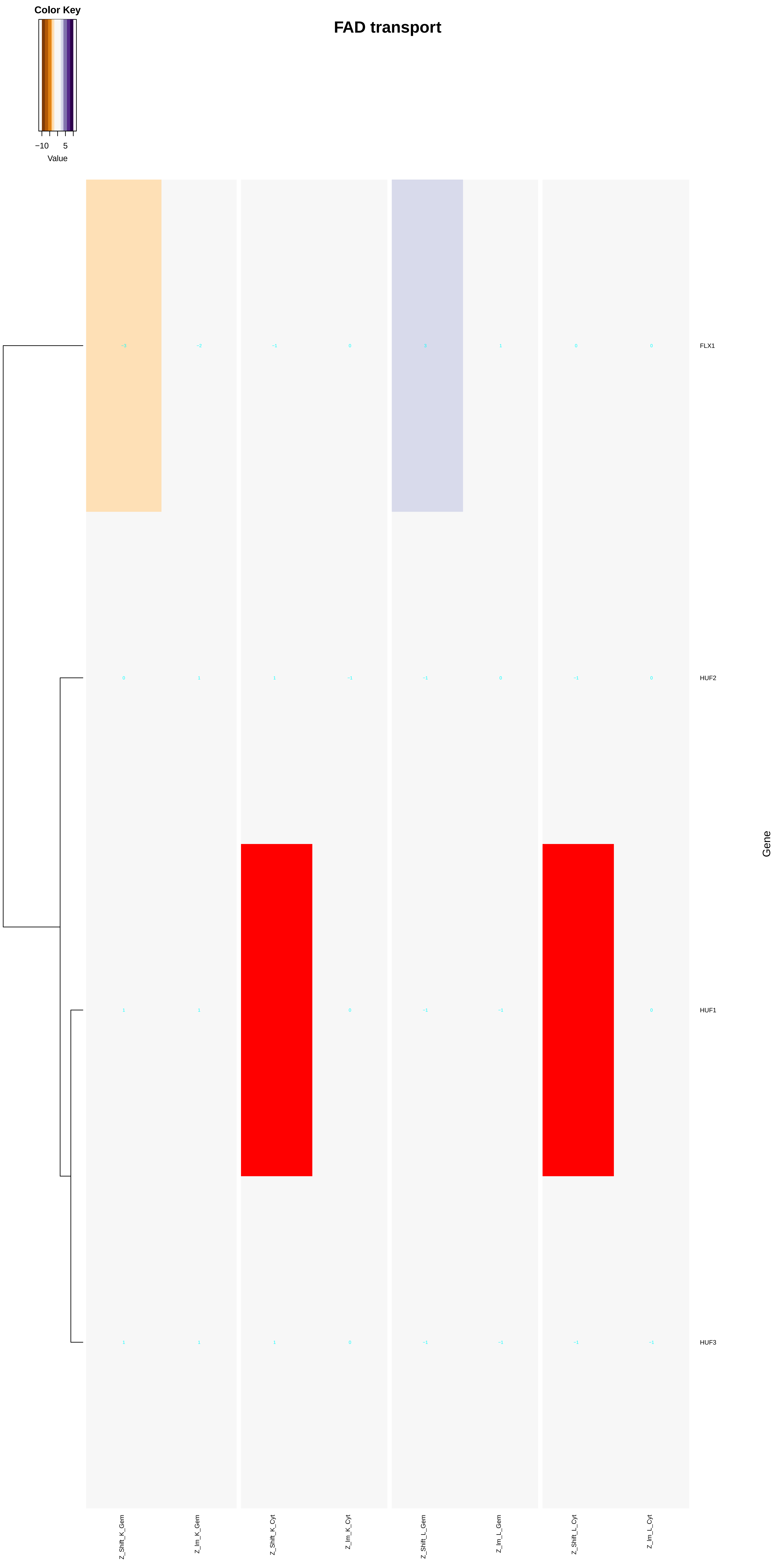

Color Key
−10
5
Value
FAD transport
FLX1
HUF2
HUF1
HUF3
Gene
Z_Shift_K_Gem
Z_Im_K_Gem
Z_Shift_K_Cyt
Z_Im_K_Cyt
Z_Shift_L_Gem
Z_Im_L_Gem
Z_Shift_L_Cyt
Z_Im_L_Cyt

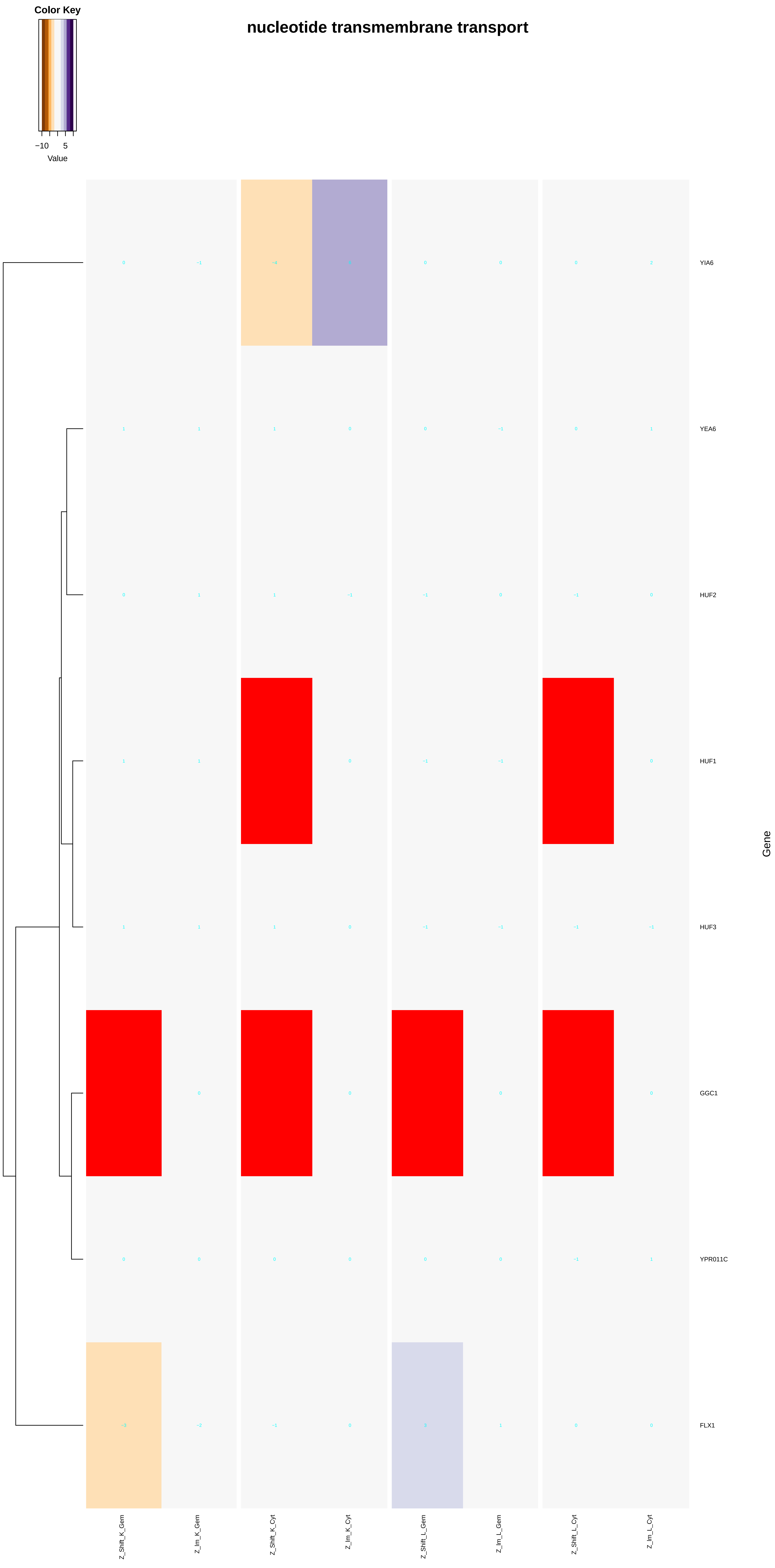
nucleotide transmembrane transport
Color Key
−10
5
Value
YIA6
YEA6
HUF2
HUF1
HUF3
GGC1
YPR011C
FLX1
Gene
Z_Shift_K_Gem
Z_Im_K_Gem
Z_Shift_K_Cyt
Z_Im_K_Cyt
Z_Shift_L_Gem
Z_Im_L_Gem
Z_Shift_L_Cyt
Z_Im_L_Cyt

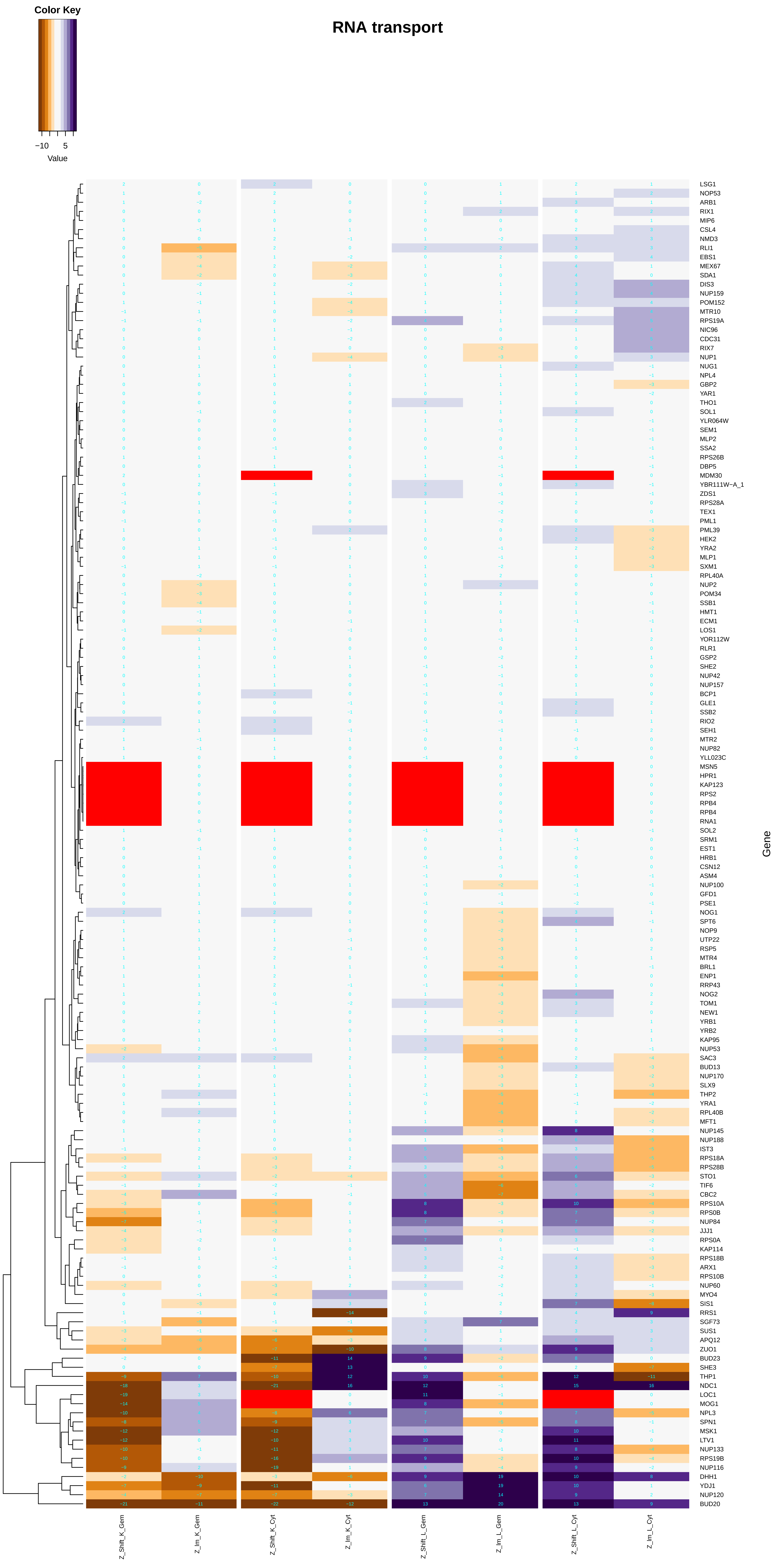

Color Key
−10
5
Value
RNA transport
LSG1
NOP53
ARB1
RIX1
MIP6
CSL4
NMD3
RLI1
EBS1
MEX67
SDA1
DIS3
NUP159
POM152
MTR10
RPS19A
NIC96
CDC31
RIX7
NUP1
NUG1
NPL4
GBP2
YAR1
THO1
SOL1
YLR064W
SEM1
MLP2
SSA2
RPS26B
DBP5
MDM30
YBR111W−A_1
ZDS1
RPS28A
TEX1
PML1
PML39
HEK2
YRA2
MLP1
SXM1
RPL40A
NUP2
POM34
SSB1
HMT1
ECM1
LOS1
YOR112W
RLR1
GSP2
SHE2
NUP42
NUP157
BCP1
GLE1
SSB2
RIO2
SEH1
MTR2
NUP82
YLL023C
MSN5
HPR1
KAP123
RPS2
RPB4
RPB4
RNA1
SOL2
SRM1
EST1
HRB1
CSN12
ASM4
NUP100
GFD1
PSE1
NOG1
SPT6
NOP9
UTP22
RSP5
MTR4
BRL1
ENP1
RRP43
NOG2
TOM1
NEW1
YRB1
YRB2
KAP95
NUP53
SAC3
BUD13
NUP170
SLX9
THP2
YRA1
RPL40B
MFT1
NUP145
NUP188
IST3
RPS18A
RPS28B
STO1
TIF6
CBC2
RPS10A
RPS0B
NUP84
JJJ1
RPS0A
KAP114
RPS18B
ARX1
RPS10B
NUP60
MYO4
SIS1
RRS1
SGF73
SUS1
APQ12
ZUO1
BUD23
SHE3
THP1
NDC1
LOC1
MOG1
NPL3
SPN1
MSK1
LTV1
NUP133
RPS19B
NUP116
DHH1
YDJ1
NUP120
BUD20
Gene
Z_Shift_K_Gem
Z_Im_K_Gem
Z_Shift_K_Cyt
Z_Im_K_Cyt
Z_Shift_L_Gem
Z_Im_L_Gem
Z_Shift_L_Cyt
Z_Im_L_Cyt

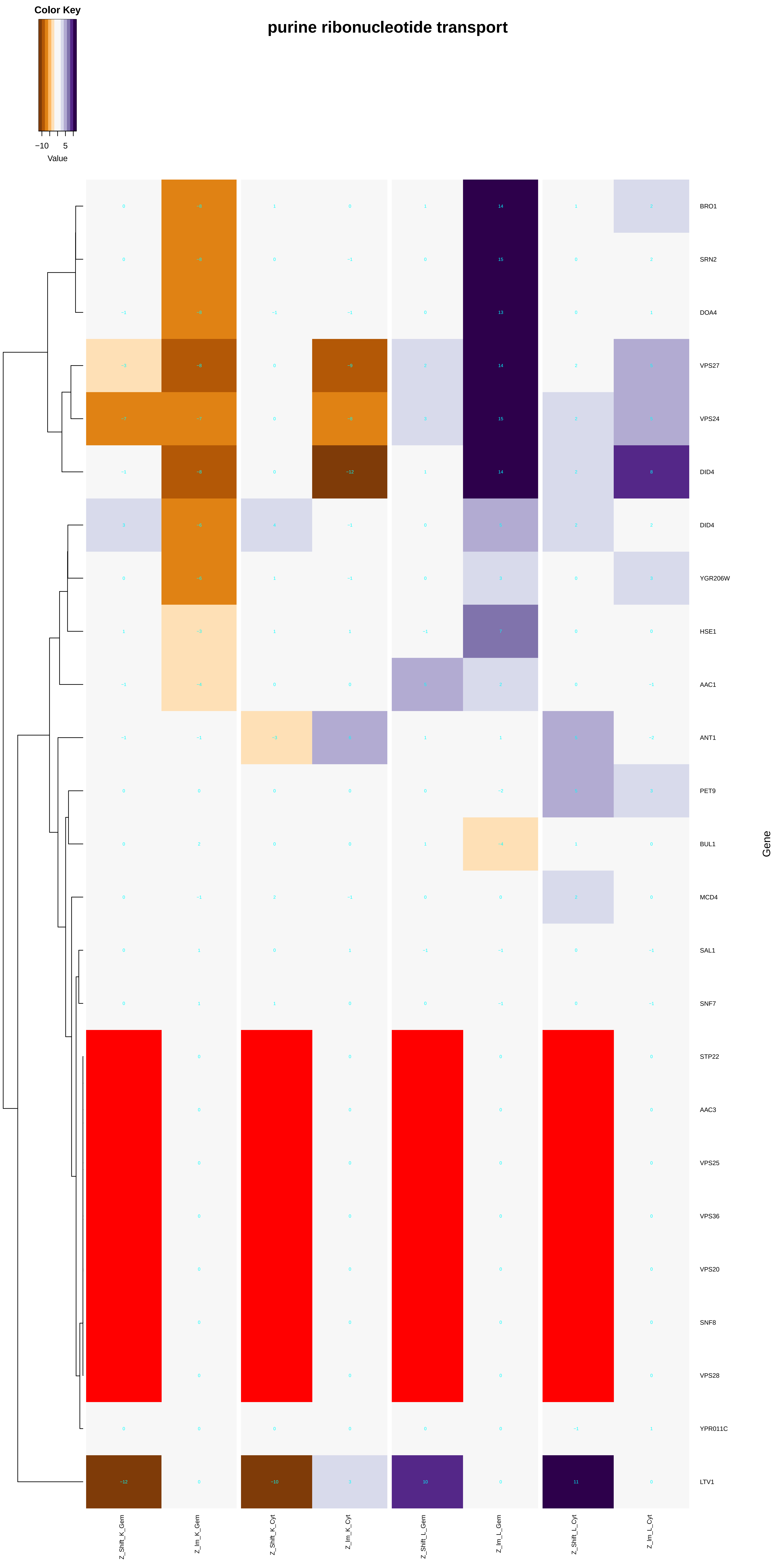
purine ribonucleotide transport
Color Key
−10
5
Value
BRO1
SRN2
DOA4
VPS27
VPS24
DID4
DID4
YGR206W
HSE1
AAC1
ANT1
PET9
BUL1
MCD4
SAL1
SNF7
STP22
AAC3
VPS25
VPS36
VPS20
SNF8
VPS28
YPR011C
LTV1
Gene
Z_Shift_K_Gem
Z_Im_K_Gem
Z_Shift_K_Cyt
Z_Im_K_Cyt
Z_Shift_L_Gem
Z_Im_L_Gem
Z_Shift_L_Cyt
Z_Im_L_Cyt

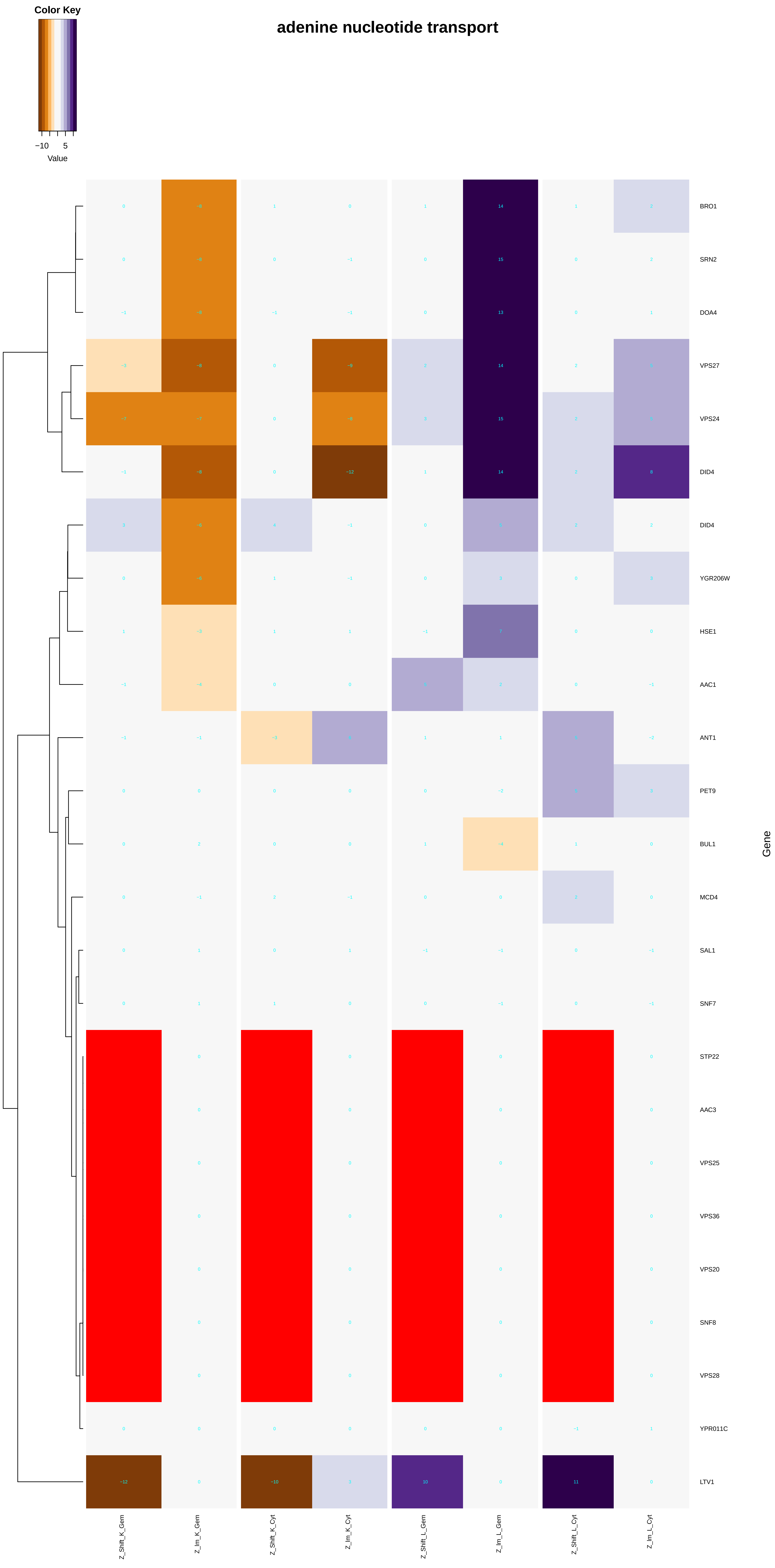
adenine nucleotide transport
Color Key
−10
5
Value
BRO1
SRN2
DOA4
VPS27
VPS24
DID4
DID4
YGR206W
HSE1
AAC1
ANT1
PET9
BUL1
MCD4
SAL1
SNF7
STP22
AAC3
VPS25
VPS36
VPS20
SNF8
VPS28
YPR011C
LTV1
Gene
Z_Shift_K_Gem
Z_Im_K_Gem
Z_Shift_K_Cyt
Z_Im_K_Cyt
Z_Shift_L_Gem
Z_Im_L_Gem
Z_Shift_L_Cyt
Z_Im_L_Cyt

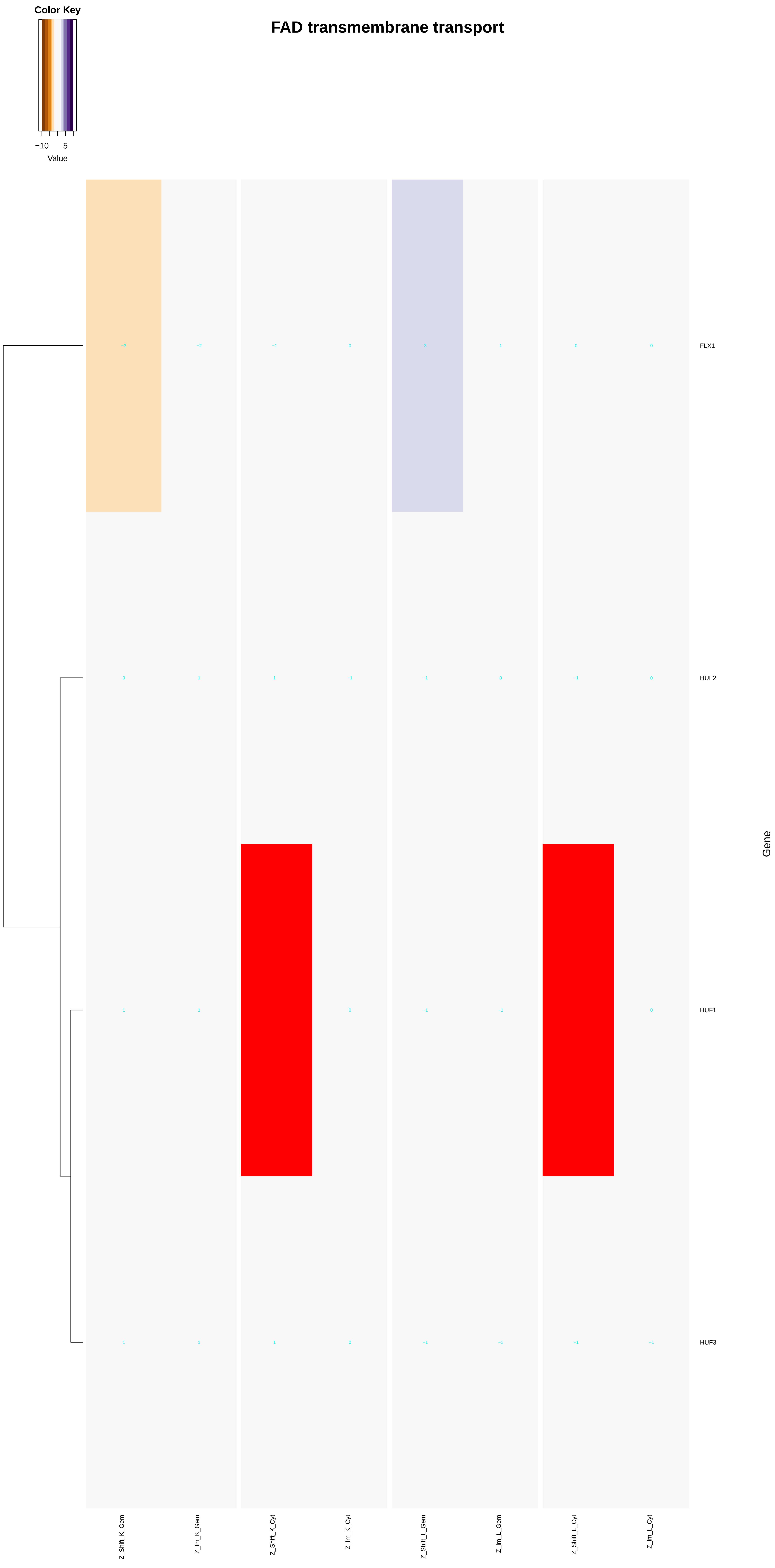

Color Key
−10
5
Value
FAD transmembrane transport
−3
−2
−1
0
3
1
0
0
FLX1
0
1
1
−1
−1
0
−1
0
HUF2
1
1
0
−1
−1
0
HUF1
1
1
1
0
−1
−1
−1
−1
HUF3
Gene
Z_Shift_K_Gem
Z_Im_K_Gem
Z_Shift_K_Cyt
Z_Im_K_Cyt
Z_Shift_L_Gem
Z_Im_L_Gem
Z_Shift_L_Cyt
Z_Im_L_Cyt

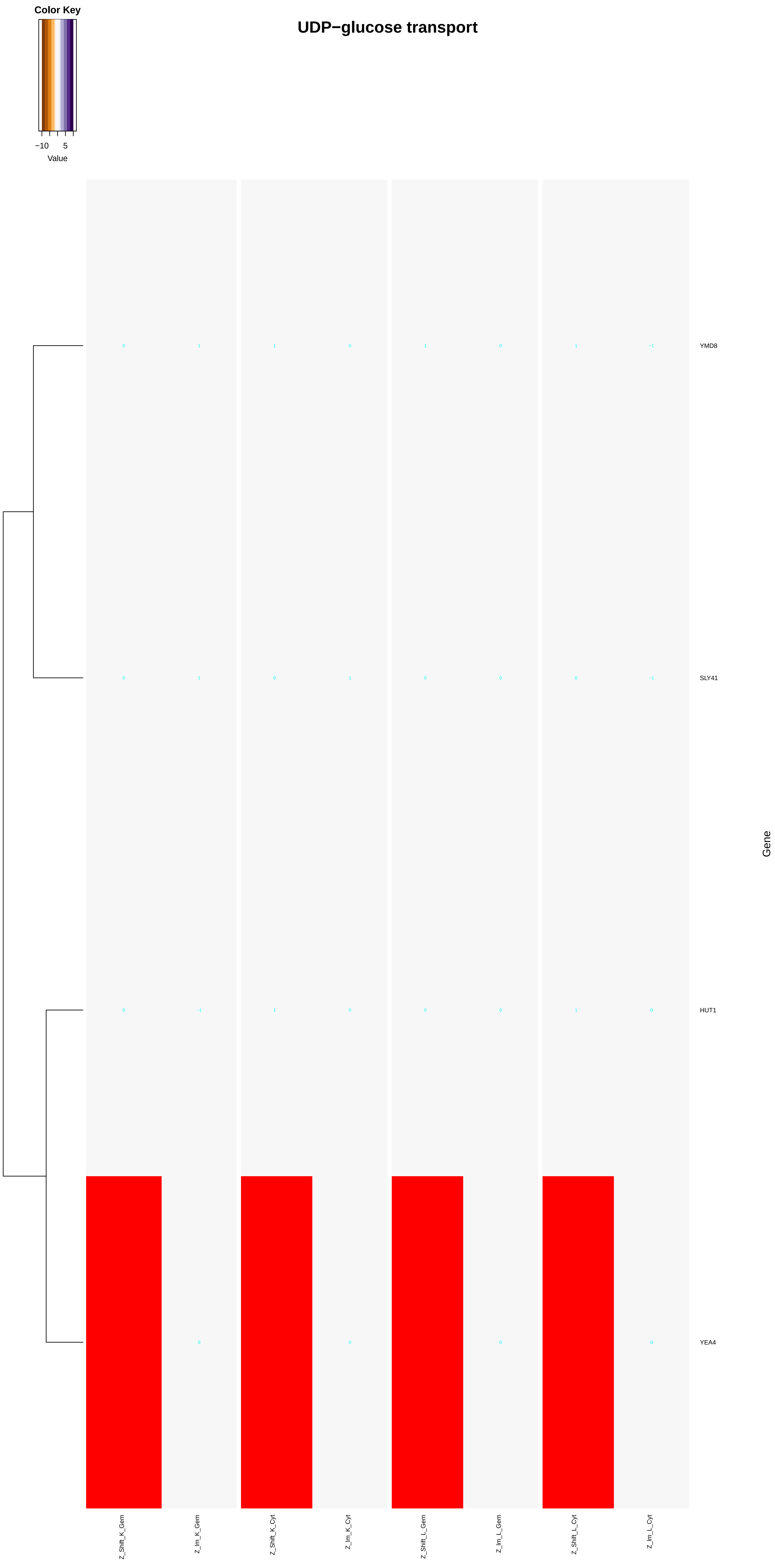

UDP−glucose transport
Color Key
−10
5
Value
YMD8
SLY41
HUT1
YEA4
Gene
Z_Shift_K_Gem
Z_Im_K_Gem
Z_Shift_K_Cyt
Z_Im_K_Cyt
Z_Shift_L_Gem
Z_Im_L_Gem
Z_Shift_L_Cyt
Z_Im_L_Cyt

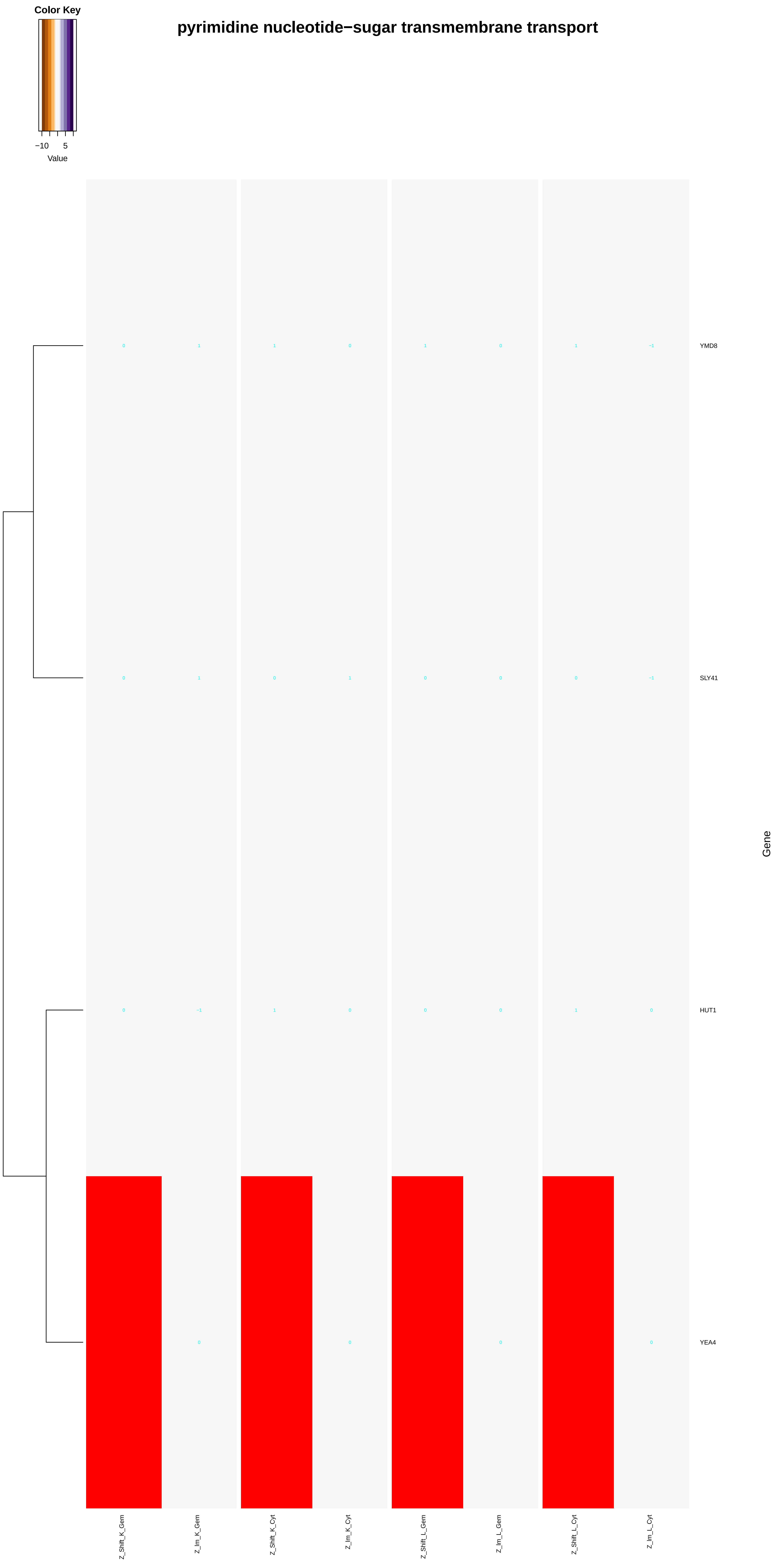

Color Key
−10
5
Value
pyrimidine nucleotide−sugar transmembrane transport
YMD8
SLY41
HUT1
YEA4
Gene
Z_Shift_K_Gem
Z_Im_K_Gem
Z_Shift_K_Cyt
Z_Im_K_Cyt
Z_Shift_L_Gem
Z_Im_L_Gem
Z_Shift_L_Cyt
Z_Im_L_Cyt

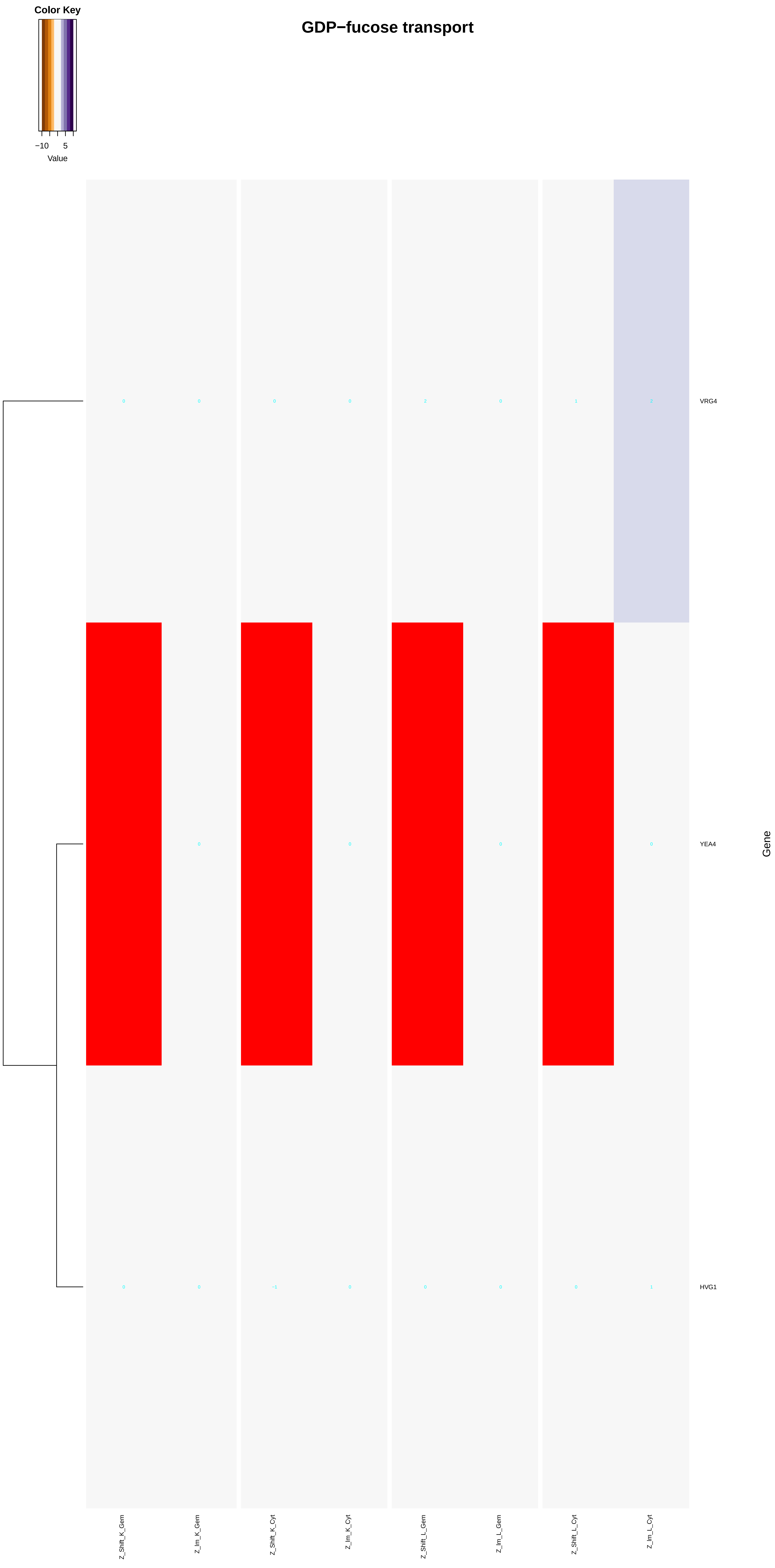

Color Key
−10
5
Value
GDP−fucose transport
VRG4
YEA4
HVG1
Gene
Z_Shift_K_Gem
Z_Im_K_Gem
Z_Shift_K_Cyt
Z_Im_K_Cyt
Z_Shift_L_Gem
Z_Im_L_Gem
Z_Shift_L_Cyt
Z_Im_L_Cyt

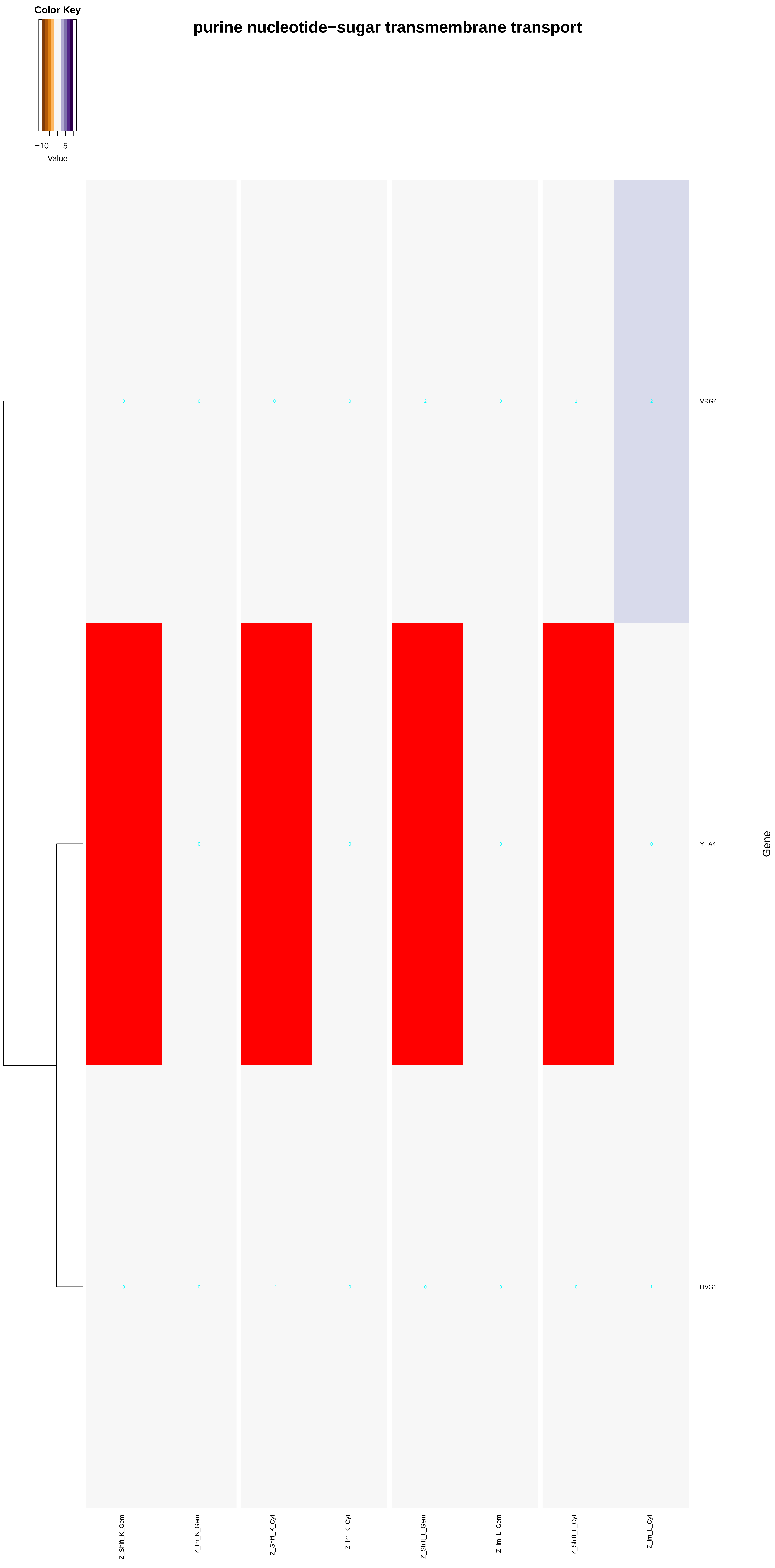

Color Key
−10
5
Value
purine nucleotide−sugar transmembrane transport
VRG4
YEA4
HVG1
Gene
Z_Shift_K_Gem
Z_Im_K_Gem
Z_Shift_K_Cyt
Z_Im_K_Cyt
Z_Shift_L_Gem
Z_Im_L_Gem
Z_Shift_L_Cyt
Z_Im_L_Cyt

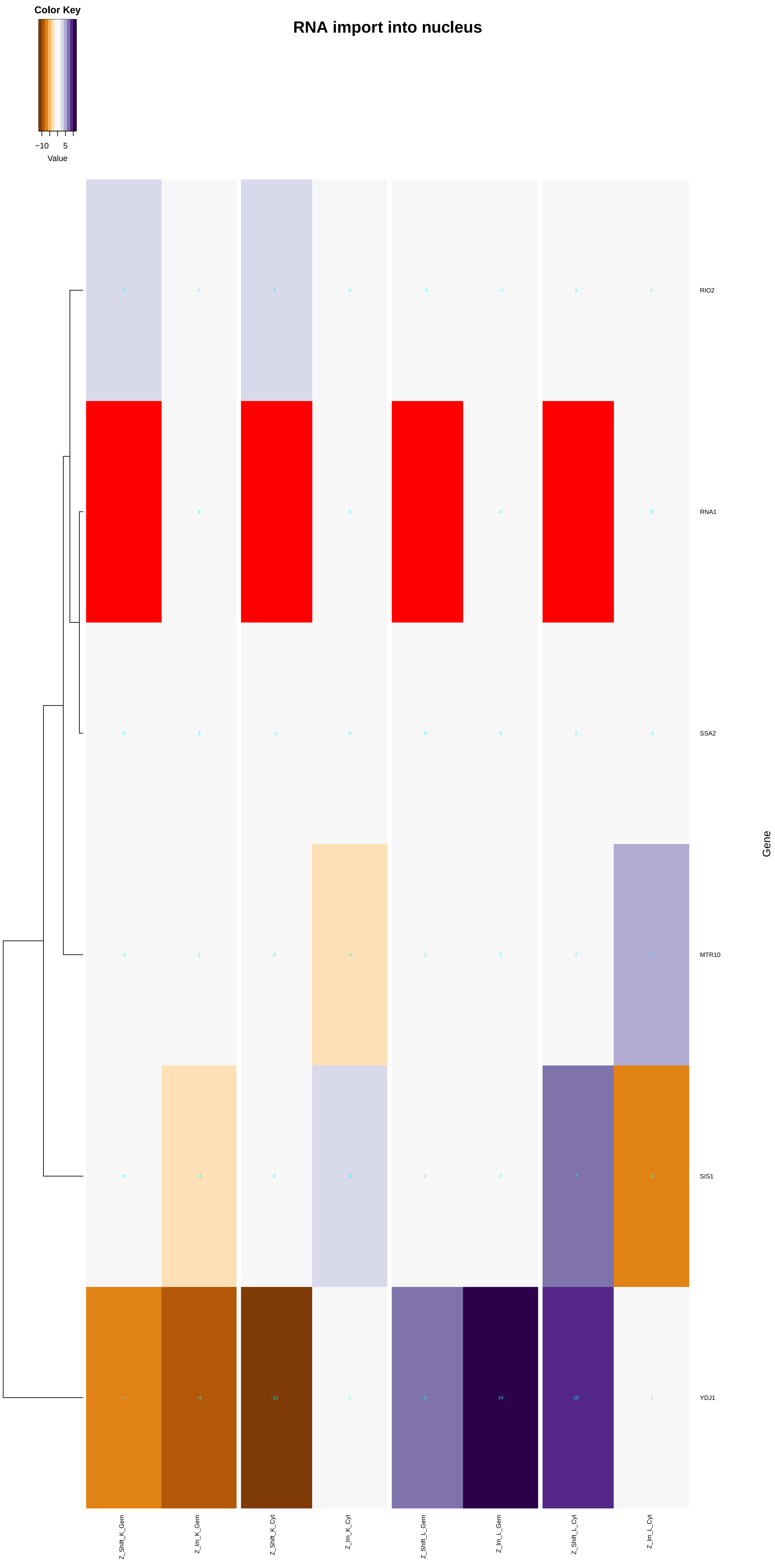
RNA import into nucleus
Color Key
−10 5
Value
RIO2
RNA1
SSA2
MTR10
SIS1
YDJ1
Gene
Z_Shift_K_Gem
Z_Im_K_Gem
Z_Shift_K_Cyt
Z_Im_K_Cyt
Z_Shift_L_Gem
Z_Im_L_Gem
Z_Shift_L_Cyt
Z_Im_L_Cyt

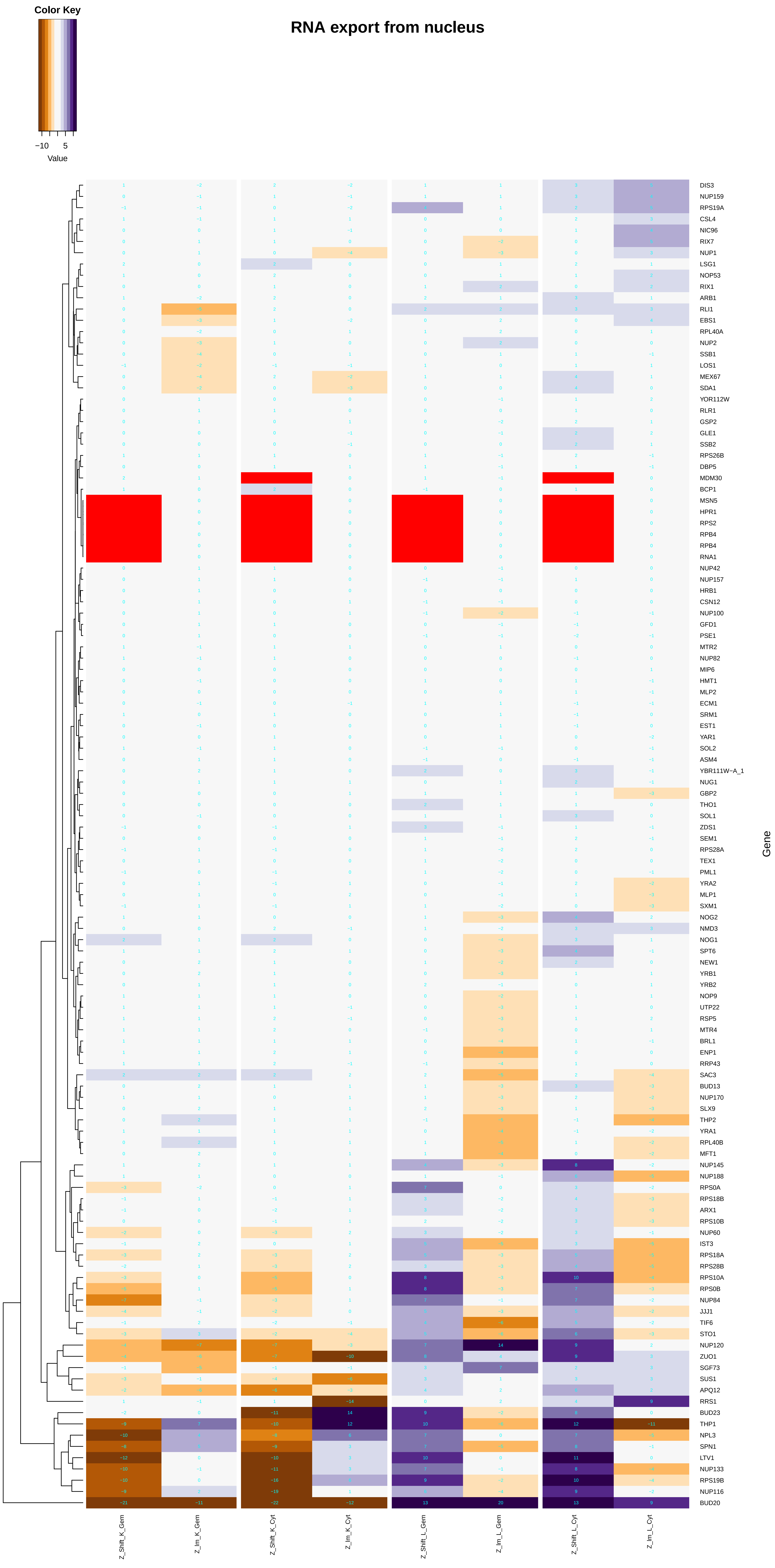

RNA export from nucleus
Color Key
−10
5
Value
DIS3
NUP159
RPS19A
CSL4
NIC96
RIX7
NUP1
LSG1
NOP53
RIX1
ARB1
RLI1
EBS1
RPL40A
NUP2
SSB1
LOS1
MEX67
SDA1
YOR112W
RLR1
GSP2
GLE1
SSB2
RPS26B
DBP5
MDM30
BCP1
MSN5
HPR1
RPS2
RPB4
RPB4
RNA1
NUP42
NUP157
HRB1
CSN12
NUP100
GFD1
PSE1
MTR2
NUP82
MIP6
HMT1
MLP2
ECM1
SRM1
EST1
YAR1
SOL2
ASM4
YBR111W−A_1
NUG1
GBP2
THO1
SOL1
ZDS1
SEM1
RPS28A
TEX1
PML1
YRA2
MLP1
SXM1
NOG2
NMD3
NOG1
SPT6
NEW1
YRB1
YRB2
NOP9
UTP22
RSP5
MTR4
BRL1
ENP1
RRP43
SAC3
BUD13
NUP170
SLX9
THP2
YRA1
RPL40B
MFT1
NUP145
NUP188
RPS0A
RPS18B
ARX1
RPS10B
NUP60
IST3
RPS18A
RPS28B
RPS10A
RPS0B
NUP84
JJJ1
TIF6
STO1
NUP120
ZUO1
SGF73
SUS1
APQ12
RRS1
BUD23
THP1
NPL3
SPN1
LTV1
NUP133
RPS19B
NUP116
BUD20
Gene
Z_Shift_K_Gem
Z_Im_K_Gem
Z_Shift_K_Cyt
Z_Im_K_Cyt
Z_Shift_L_Gem
Z_Im_L_Gem
Z_Shift_L_Cyt
Z_Im_L_Cyt

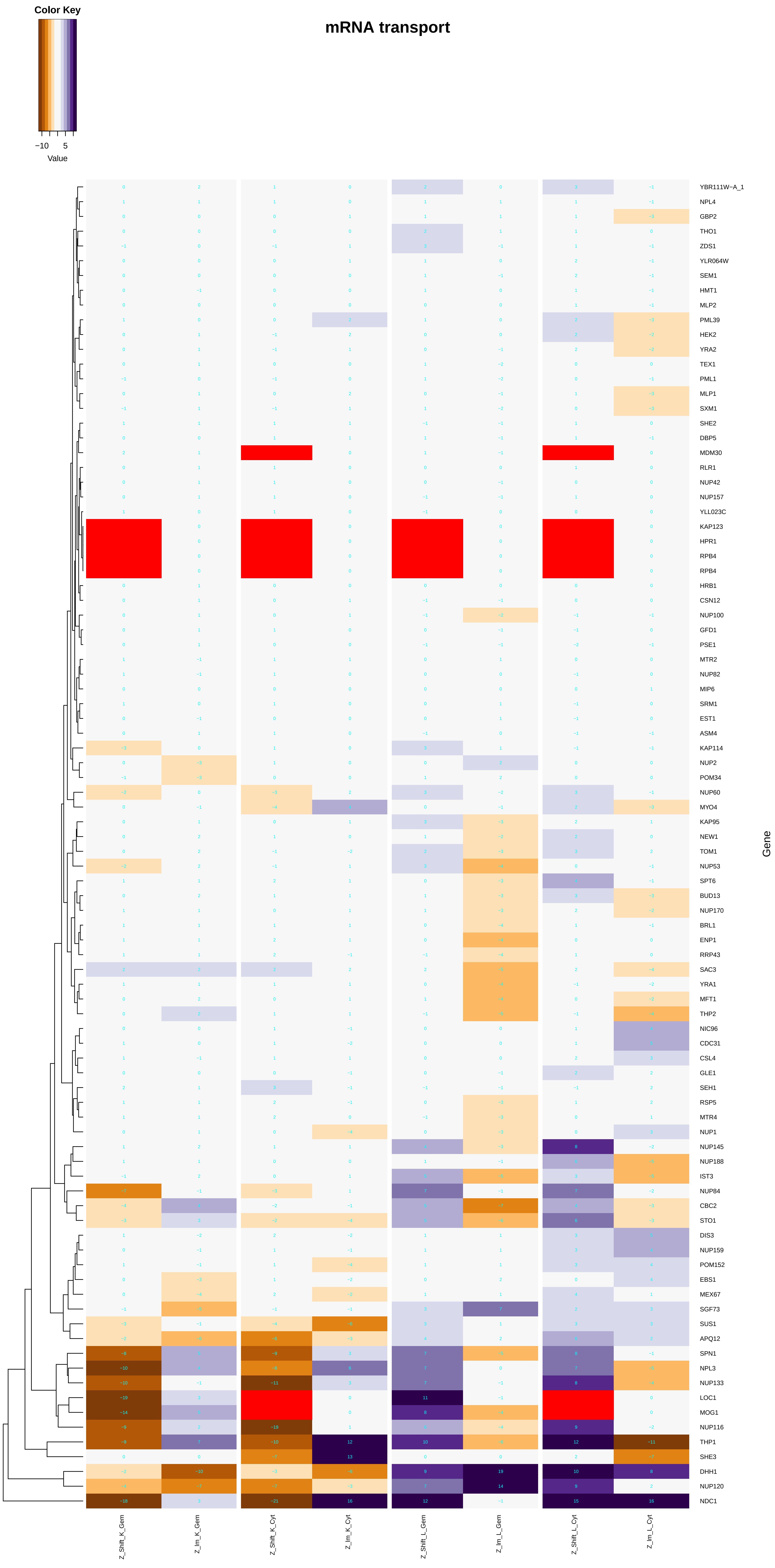
Color Key
−10
5
Value
mRNA transport
YBR111W−A_1
NPL4
GBP2
THO1
ZDS1
YLR064W
SEM1
HMT1
MLP2
PML39
HEK2
YRA2
TEX1
PML1
MLP1
SXM1
SHE2
DBP5
MDM30
RLR1
NUP42
NUP157
YLL023C
KAP123
HPR1
RPB4
RPB4
HRB1
CSN12
NUP100
GFD1
PSE1
MTR2
NUP82
MIP6
SRM1
EST1
ASM4
KAP114
NUP2
POM34
NUP60
MYO4
KAP95
NEW1
TOM1
NUP53
SPT6
BUD13
NUP170
BRL1
ENP1
RRP43
SAC3
YRA1
MFT1
THP2
NIC96
CDC31
CSL4
GLE1
SEH1
RSP5
MTR4
NUP1
NUP145
NUP188
IST3
NUP84
CBC2
STO1
DIS3
NUP159
POM152
EBS1
MEX67
SGF73
SUS1
APQ12
SPN1
NPL3
NUP133
LOC1
MOG1
NUP116
THP1
SHE3
DHH1
NUP120
NDC1
Gene
Z_Shift_K_Gem
Z_Im_K_Gem
Z_Shift_K_Cyt
Z_Im_K_Cyt
Z_Shift_L_Gem
Z_Im_L_Gem
Z_Shift_L_Cyt
Z_Im_L_Cyt

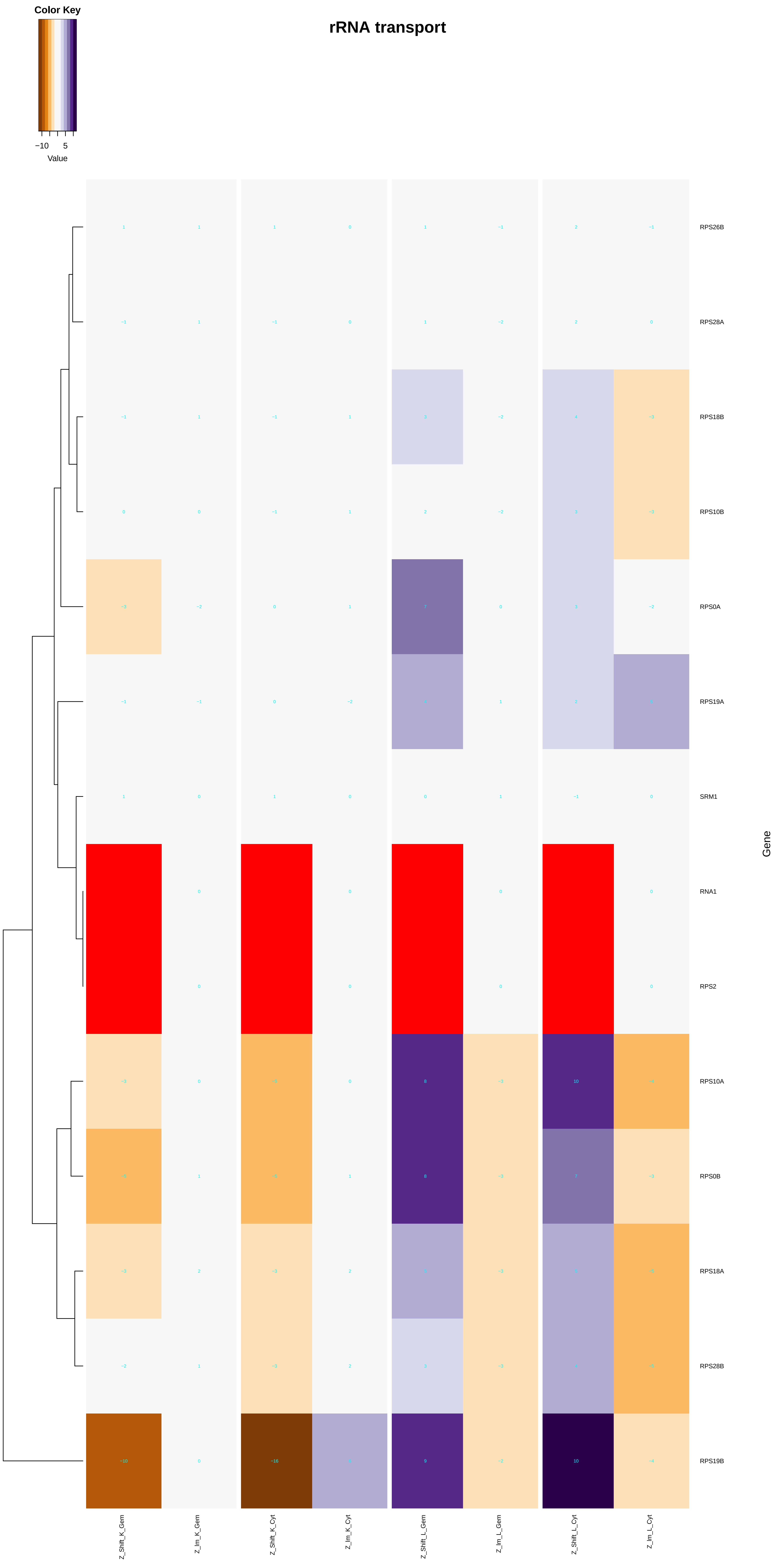
rRNA transport
Color Key
−10
5
Value
Gene
RPS26B
RPS28A
RPS18B
RPS10B
RPS0A
RPS19A
SRM1
RNA1
RPS2
RPS10A
RPS0B
RPS18A
RPS28B
RPS19B
Z_Shift_K_Gem
Z_Im_K_Gem
Z_Shift_K_Cyt
Z_Im_K_Cyt
Z_Shift_L_Gem
Z_Im_L_Gem
Z_Shift_L_Cyt
Z_Im_L_Cyt

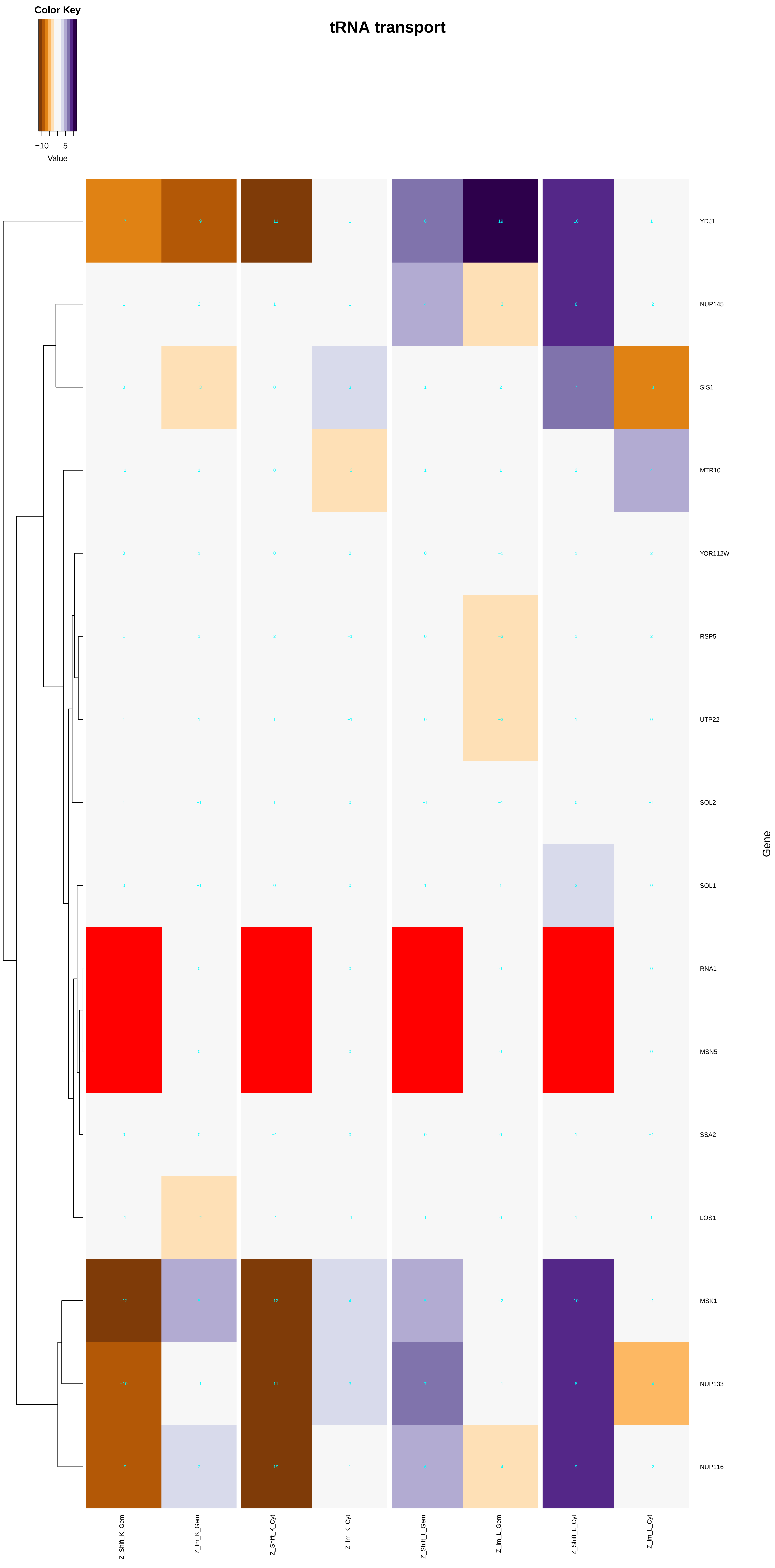
Color Key
−10
5
Value
tRNA transport
YDJ1
NUP145
SIS1
MTR10
YOR112W
RSP5
UTP22
SOL2
SOL1
RNA1
MSN5
SSA2
LOS1
MSK1
NUP133
NUP116
Gene
Z_Shift_K_Gem
Z_Im_K_Gem
Z_Shift_K_Cyt
Z_Im_K_Cyt
Z_Shift_L_Gem
Z_Im_L_Gem
Z_Shift_L_Cyt
Z_Im_L_Cyt

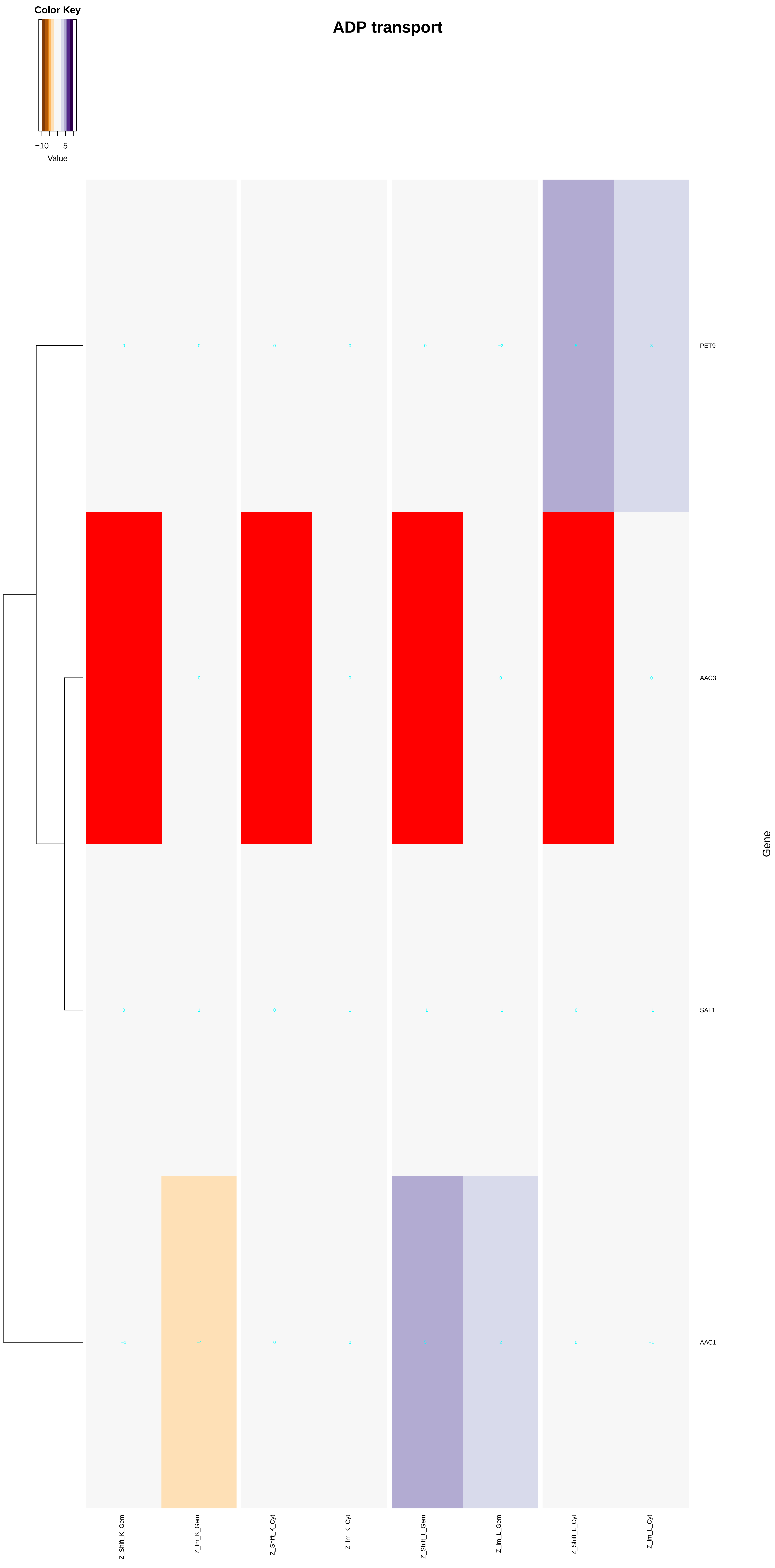

Color Key
−10
5
Value
ADP transport
PET9
AAC3
SAL1
AAC1
Gene
Z_Shift_K_Gem
Z_Im_K_Gem
Z_Shift_K_Cyt
Z_Im_K_Cyt
Z_Shift_L_Gem
Z_Im_L_Gem
Z_Shift_L_Cyt
Z_Im_L_Cyt

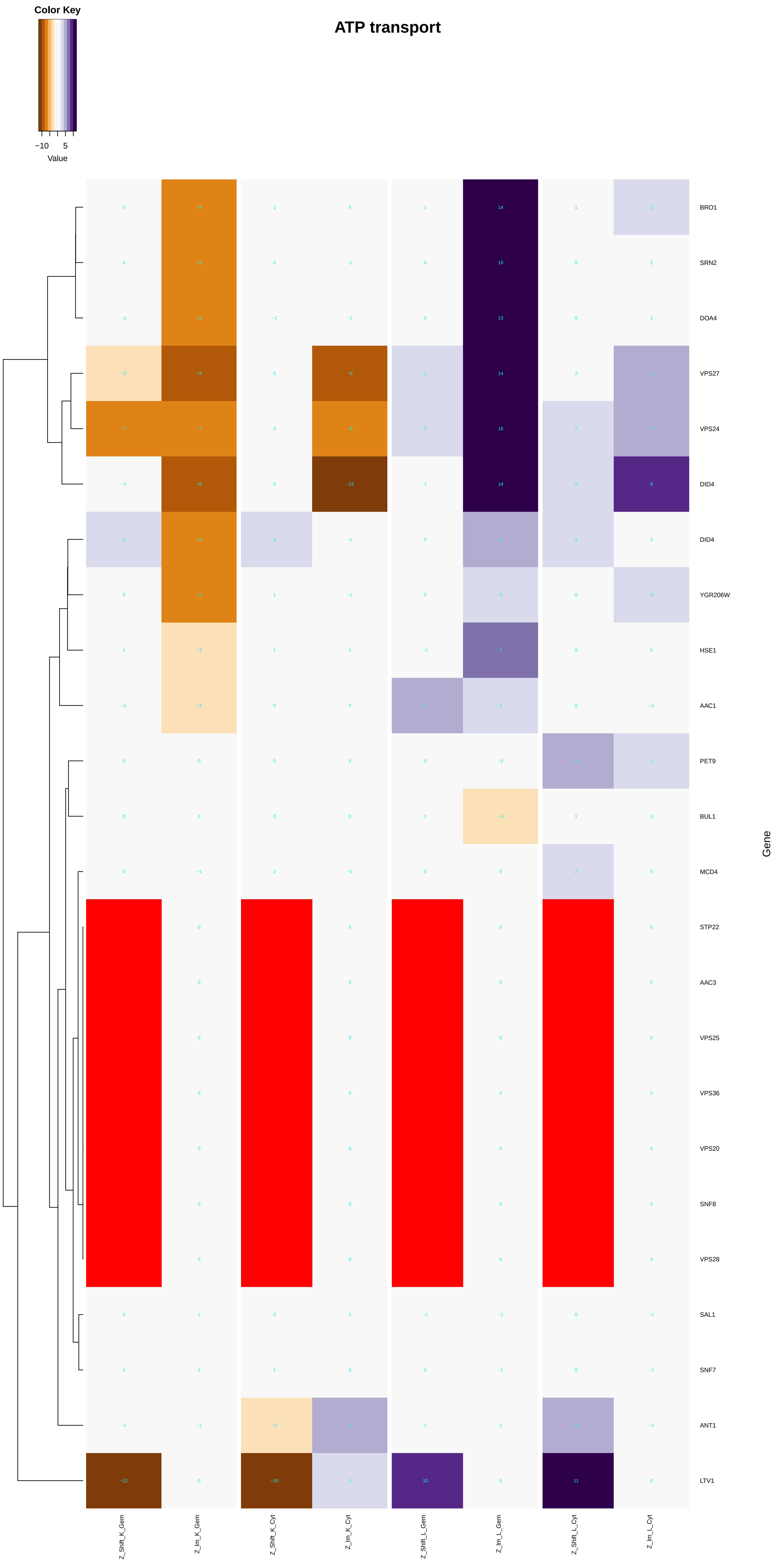
ATP transport
Color Key
−10
5
Value
BRO1
SRN2
DOA4
VPS27
VPS24
DID4
DID4
YGR206W
HSE1
AAC1
PET9
BUL1
MCD4
STP22
AAC3
VPS25
VPS36
VPS20
SNF8
VPS28
SAL1
SNF7
ANT1
LTV1
Gene
Z_Shift_K_Gem
Z_Im_K_Gem
Z_Shift_K_Cyt
Z_Im_K_Cyt
Z_Shift_L_Gem
Z_Im_L_Gem
Z_Shift_L_Cyt
Z_Im_L_Cyt

tRNA import into nucleus

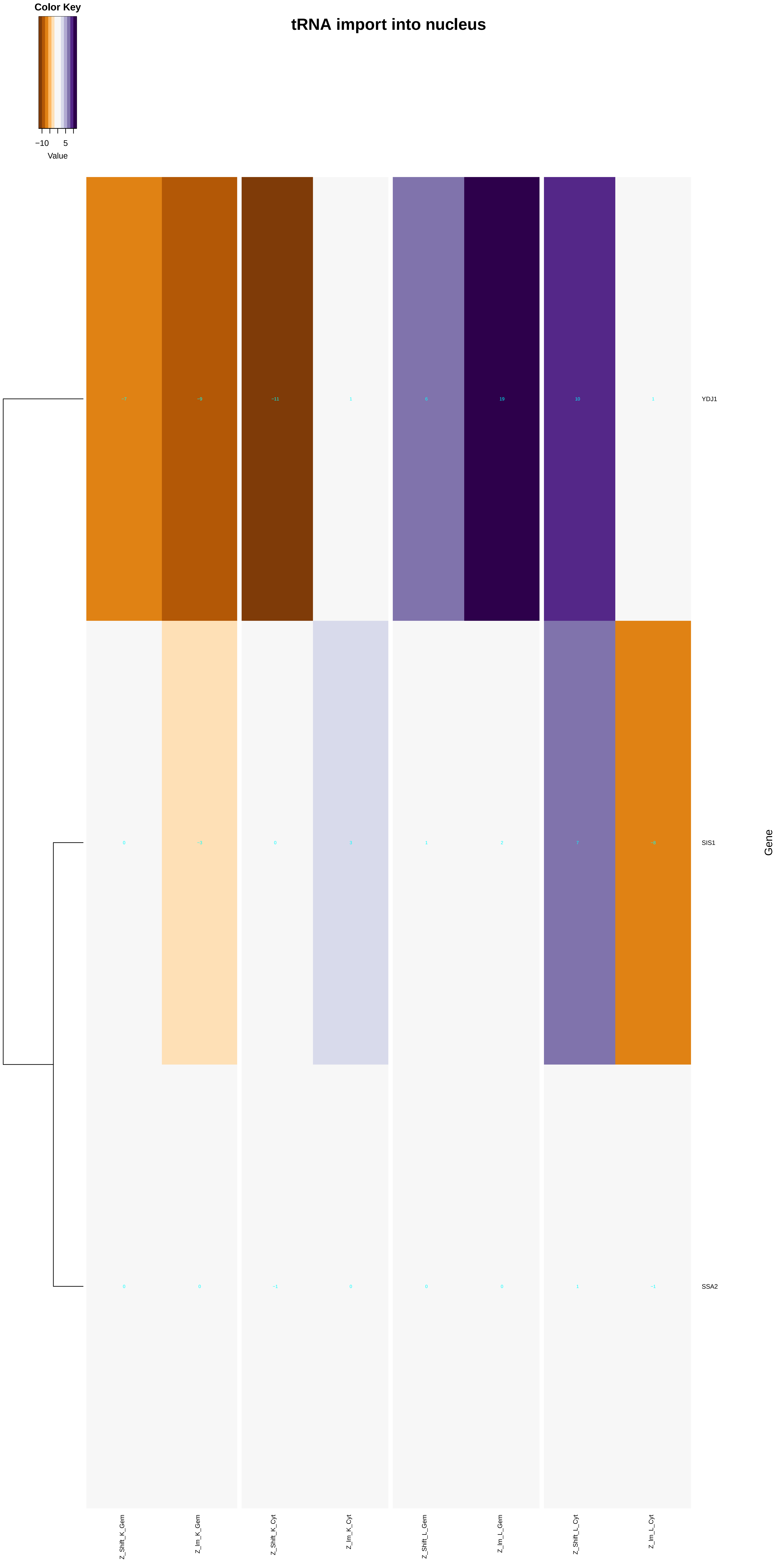

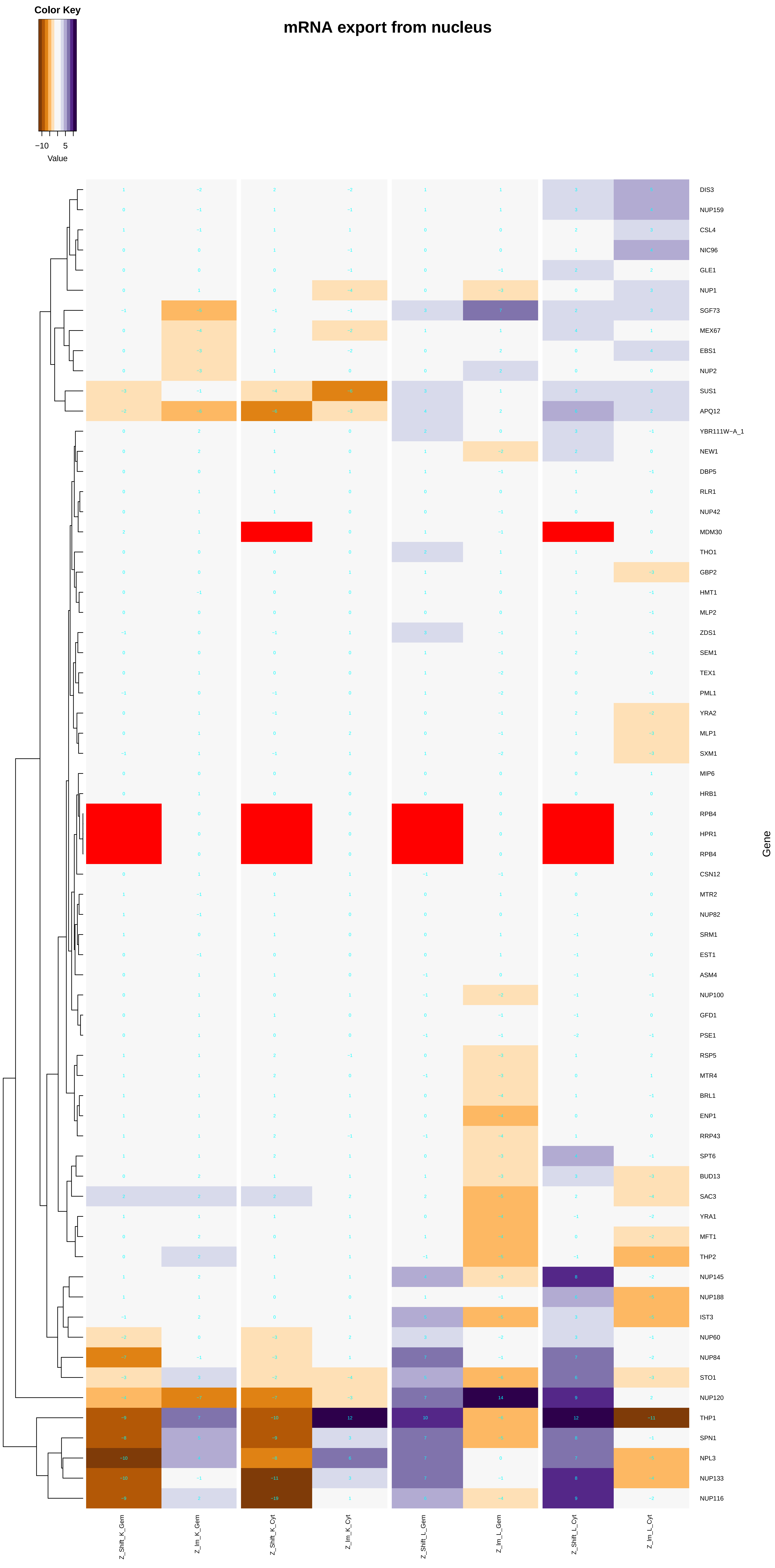

mRNA export from nucleus
Color Key
−10
5
Value
Gene
DIS3
NUP159
CSL4
NIC96
GLE1
NUP1
SGF73
MEX67
EBS1
NUP2
SUS1
APQ12
YBR111W−A_1
NEW1
DBP5
RLR1
NUP42
MDM30
THO1
GBP2
HMT1
MLP2
ZDS1
SEM1
TEX1
PML1
YRA2
MLP1
SXM1
MIP6
HRB1
RPB4
HPR1
RPB4
CSN12
MTR2
NUP82
SRM1
EST1
ASM4
NUP100
GFD1
PSE1
RSP5
MTR4
BRL1
ENP1
RRP43
SPT6
BUD13
SAC3
YRA1
MFT1
THP2
NUP145
NUP188
IST3
NUP60
NUP84
STO1
NUP120
THP1
SPN1
NPL3
NUP133
NUP116
Z_Shift_K_Gem
Z_Im_K_Gem
Z_Shift_K_Cyt
Z_Im_K_Cyt
Z_Shift_L_Gem
Z_Im_L_Gem
Z_Shift_L_Cyt
Z_Im_L_Cyt

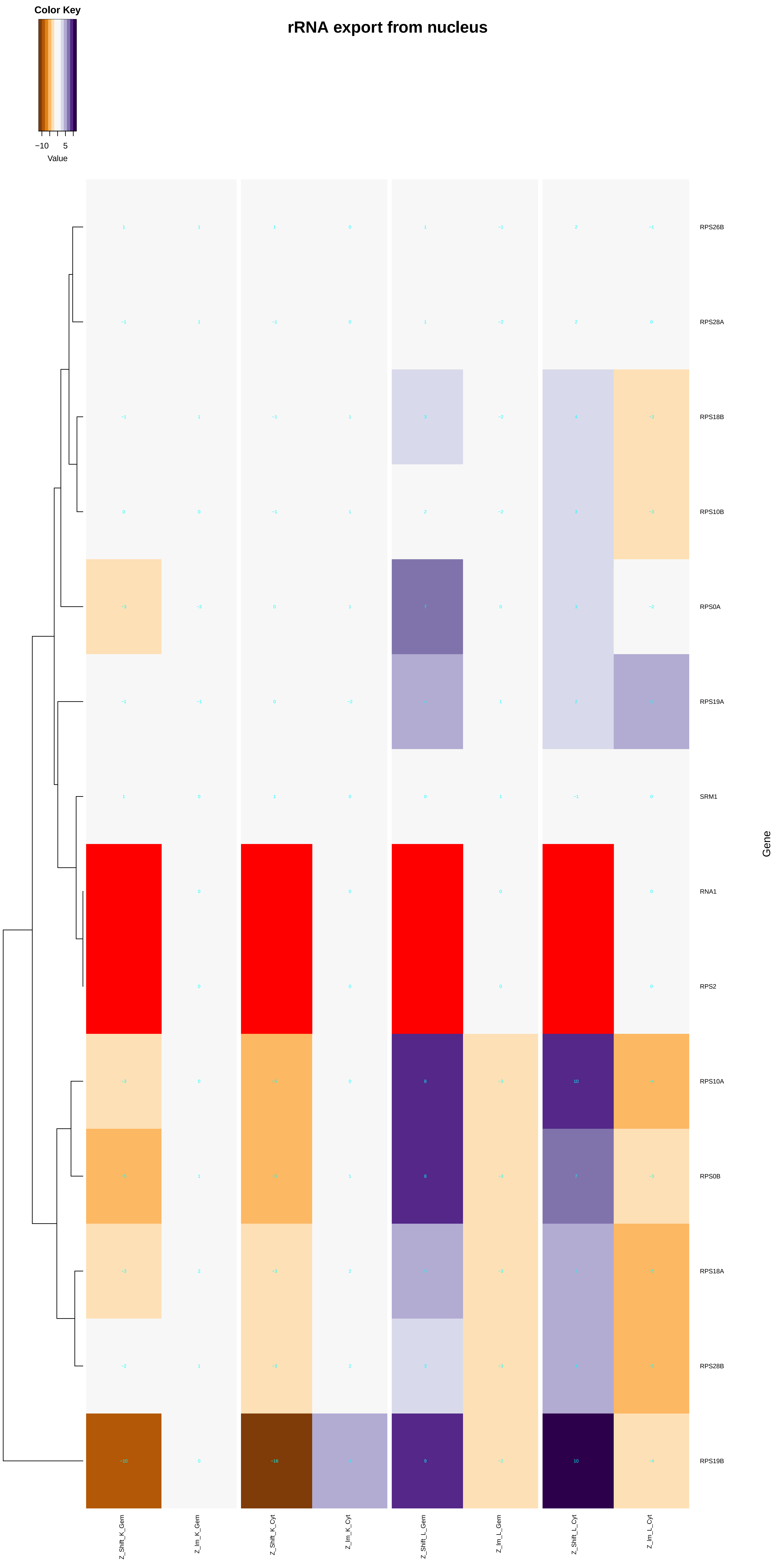
Color Key
−10
5
Value
rRNA export from nucleus
RPS26B
RPS28A
RPS18B
RPS10B
RPS0A
RPS19A
SRM1
RNA1
RPS2
RPS10A
RPS0B
RPS18A
RPS28B
RPS19B
Gene
Z_Shift_K_Gem
Z_Im_K_Gem
Z_Shift_K_Cyt
Z_Im_K_Cyt
Z_Shift_L_Gem
Z_Im_L_Gem
Z_Shift_L_Cyt
Z_Im_L_Cyt

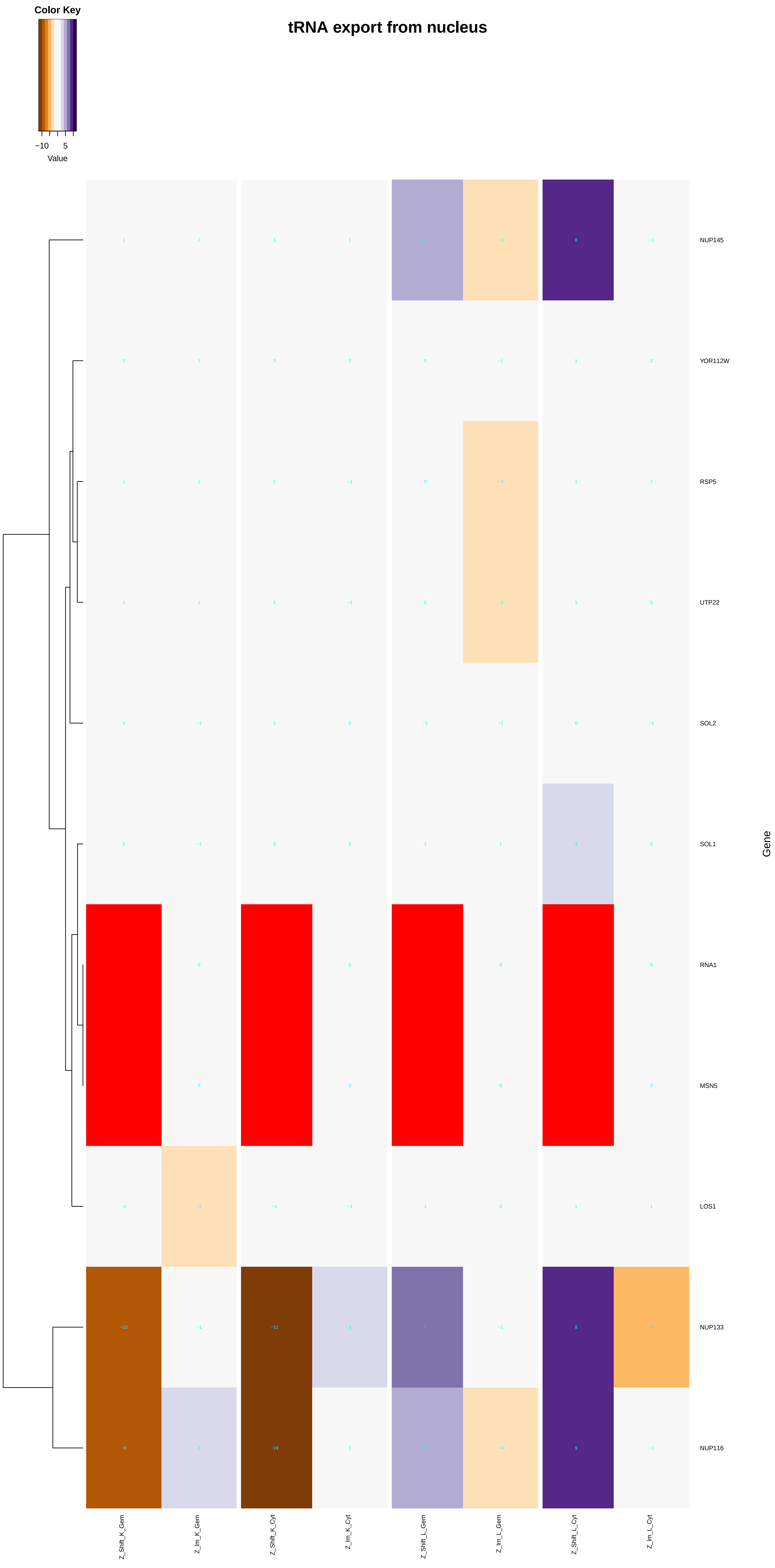
Color Key
−10
5
Value
tRNA export from nucleus
NUP145
YOR112W
RSP5
UTP22
SOL2
SOL1
RNA1
MSN5
LOS1
NUP133
NUP116
Gene
Z_Shift_K_Gem
Z_Im_K_Gem
Z_Shift_K_Cyt
Z_Im_K_Cyt
Z_Shift_L_Gem
Z_Im_L_Gem
Z_Shift_L_Cyt
Z_Im_L_Cyt

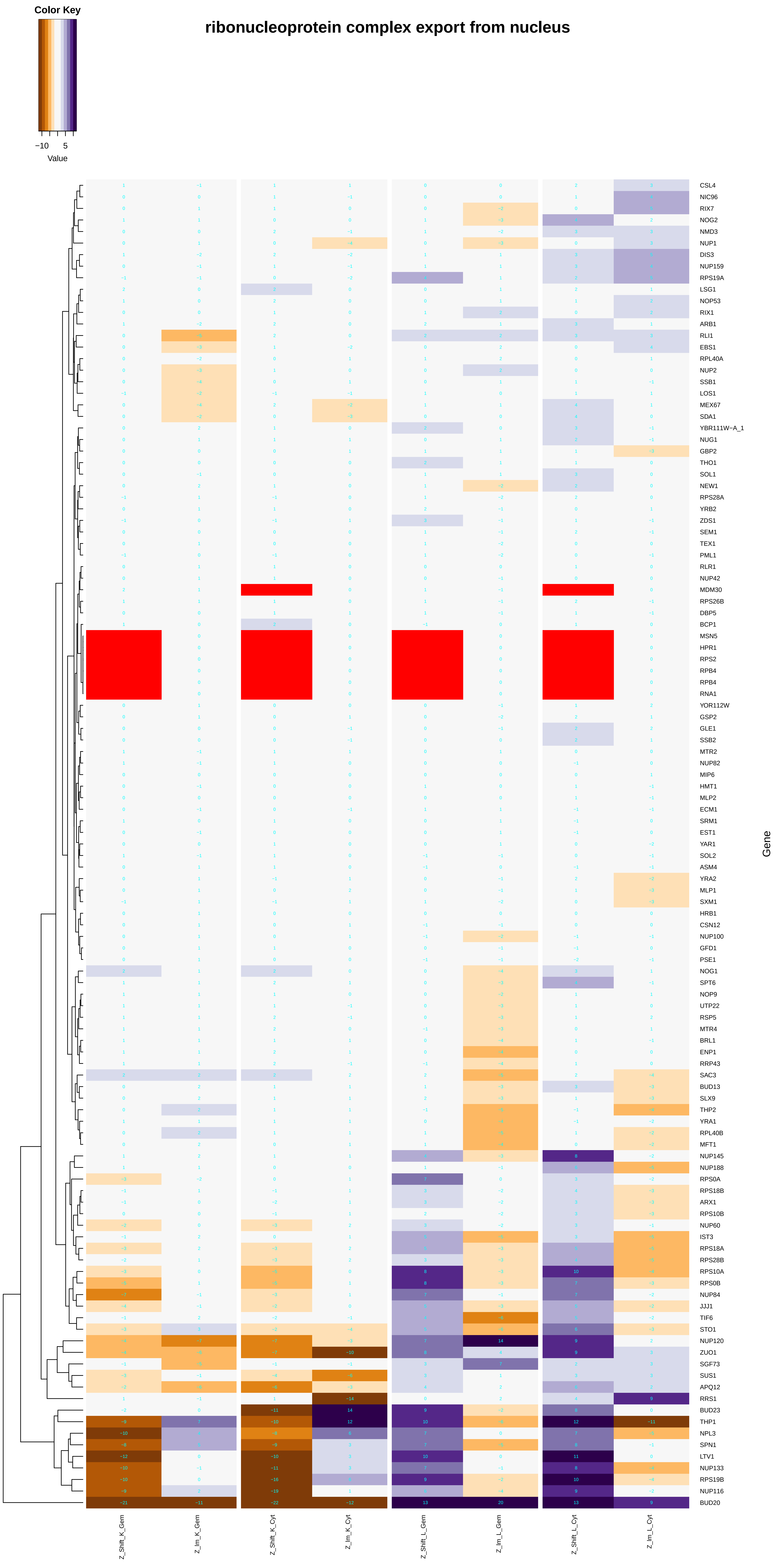
Color Key
−10 5
Value
ribonucleoprotein complex export from nucleus
CSL4
NIC96
RIX7
NOG2
NMD3
NUP1
DIS3
NUP159
RPS19A
LSG1
NOP53
RIX1
ARB1
RLI1
EBS1
RPL40A
NUP2
SSB1
LOS1
MEX67
SDA1
YBR111W-A_1
NUG1
GBP2
THO1
SOL1
NEW1
RPS28A
YRB2
ZDS1
SEM1
TEX1
PML1
RLR1
NUP42
MDM30
RPS26B
DBP5
BCP1
MSN5
HPR1
RPS2
RPB4
RPB4
RNA1
YOR112W
GSP2
GLE1
SSB2
MTR2
NUP82
MIP6
HMT1
MLP2
ECM1
SRM1
EST1
YAR1
SOL2
ASM4
YRA2
MLP1
SXM1
HRB1
CSN12
NUP100
GFD1
PSE1
NOG1
SPT6
NOP9
UTP22
RSP5
MTR4
BRL1
ENP1
RRP43
SAC3
BUD13
SLX9
THP2
YRA1
RPL40B
MFT1
NUP145
NUP188
RPS0A
RPS18B
ARX1
RPS10B
NUP60
IST3
RPS18A
RPS28B
RPS10A
RPS0B
NUP84
JJJ1
TIF6
STO1
NUP120
ZUO1
SGF73
SUS1
APQ12
RRS1
BUD23
THP1
NPL3
SPN1
LTV1
NUP133
RPS19B
NUP116
BUD20
Gene
Z_Shift_K_Gem
Z_Im_K_Gem
Z_Shift_K_Cyt
Z_Im_K_Cyt
Z_Shift_L_Gem
Z_Im_L_Gem
Z_Shift_L_Cyt
Z_Im_L_Cyt

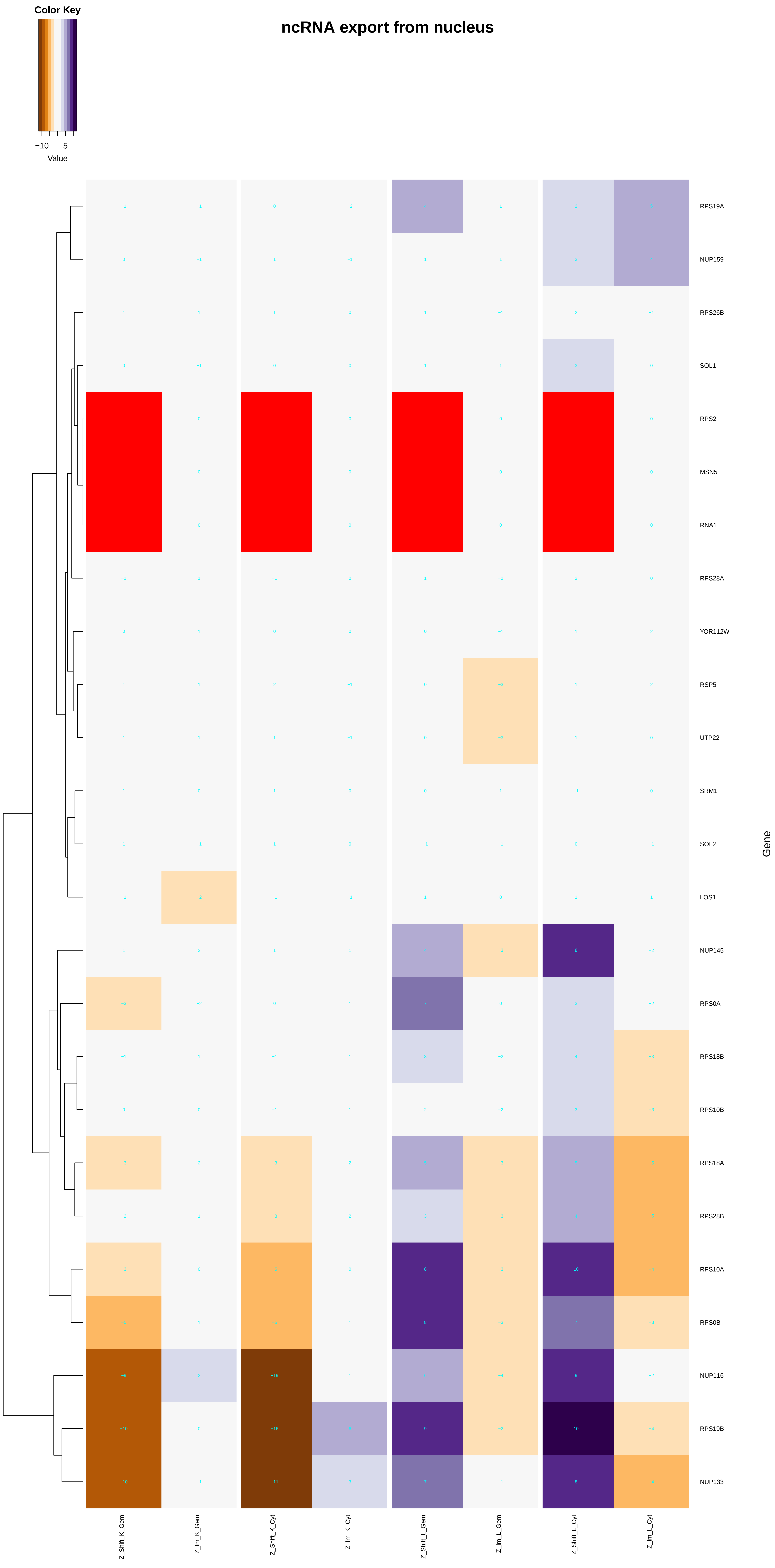

ncRNA export from nucleus
Color Key
−10
5
Value
RPS19A
NUP159
RPS26B
SOL1
RPS2
MSN5
RNA1
RPS28A
YOR112W
RSP5
UTP22
SRM1
SOL2
LOS1
NUP145
RPS0A
RPS18B
RPS10B
RPS18A
RPS28B
RPS10A
RPS0B
NUP116
RPS19B
NUP133
Gene
Z_Shift_K_Gem
Z_Im_K_Gem
Z_Shift_K_Cyt
Z_Im_K_Cyt
Z_Shift_L_Gem
Z_Im_L_Gem
Z_Shift_L_Cyt
Z_Im_L_Cyt

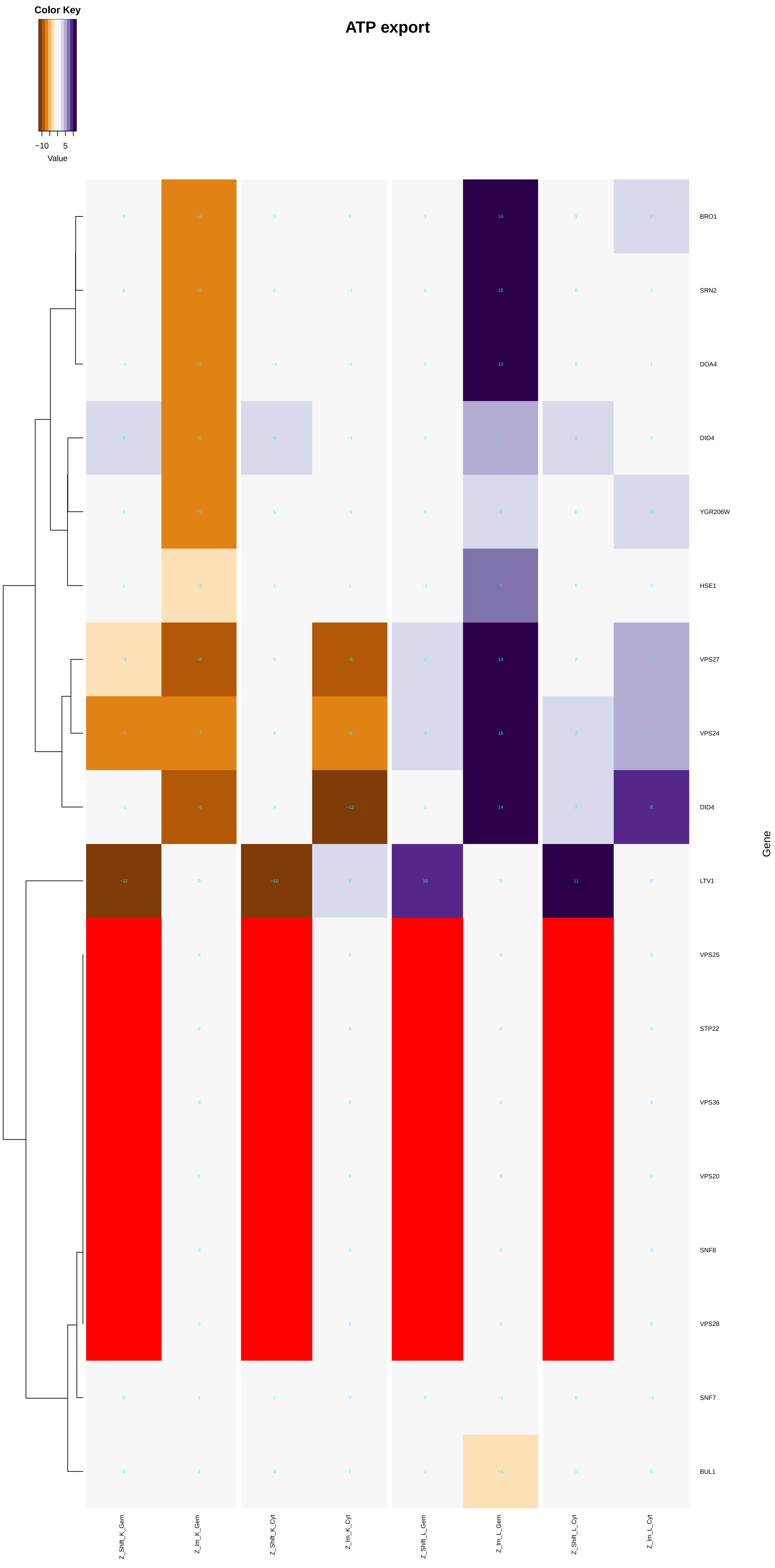

Color Key
−10
5
Value
ATP export
BRO1
SRN2
DOA4
DID4
YGR206W
HSE1
VPS27
VPS24
DID4
LTV1
VPS25
STP22
VPS36
VPS20
SNF8
VPS28
SNF7
BUL1
Gene
Z_Shift_K_Gem
Z_Im_K_Gem
Z_Shift_K_Cyt
Z_Im_K_Cyt
Z_Shift_L_Gem
Z_Im_L_Gem
Z_Shift_L_Cyt
Z_Im_L_Cyt

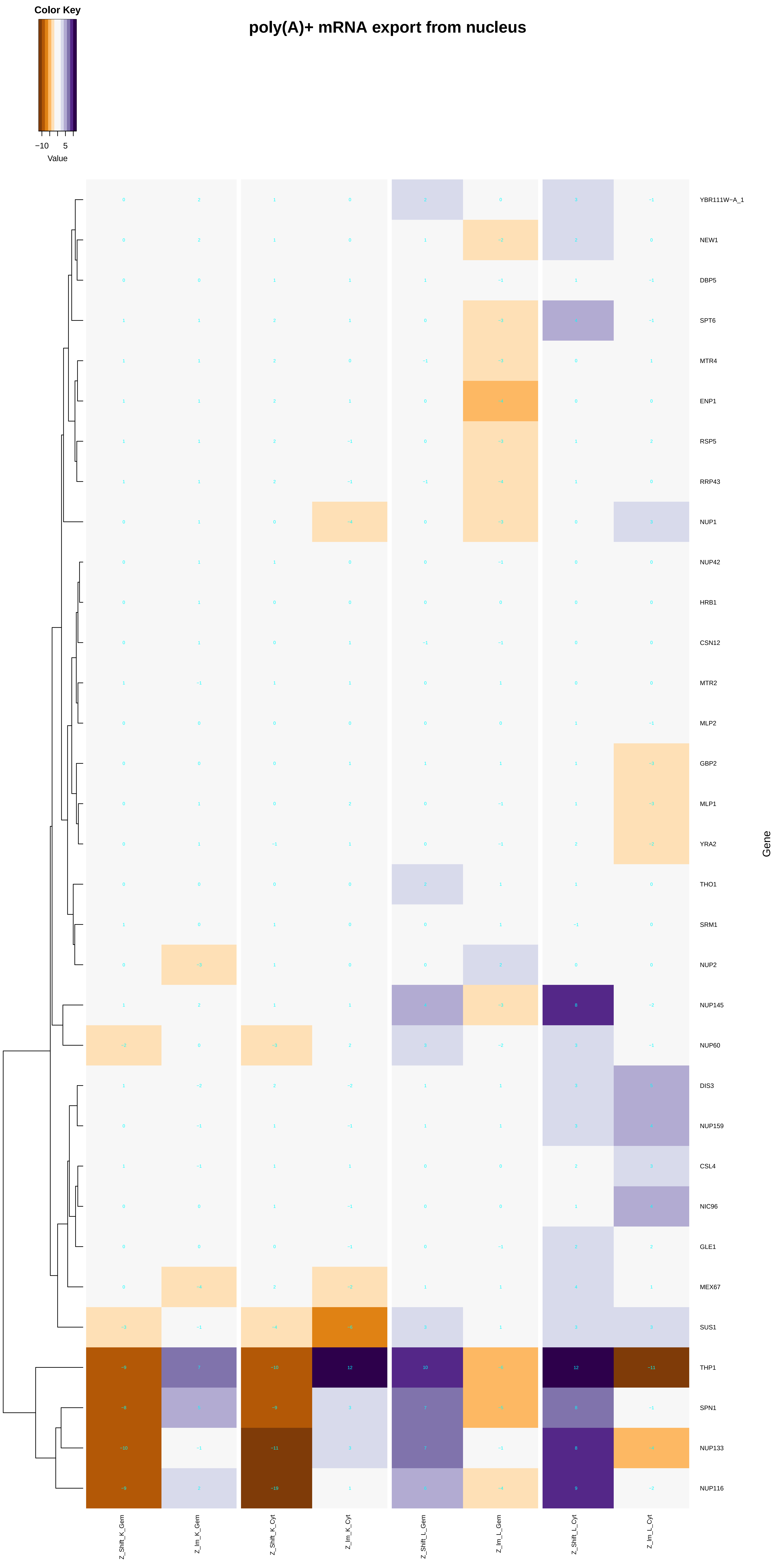
Color Key
−10
5
Value
poly(A)+ mRNA export from nucleus
YBR111W−A_1
NEW1
DBP5
SPT6
MTR4
ENP1
RSP5
RRP43
NUP1
NUP42
HRB1
CSN12
MTR2
MLP2
GBP2
MLP1
YRA2
THO1
SRM1
NUP2
NUP145
NUP60
DIS3
NUP159
CSL4
NIC96
GLE1
MEX67
SUS1
THP1
SPN1
NUP133
NUP116
Gene
Z_Shift_K_Gem
Z_lm_K_Gem
Z_Shift_K_Cyt
Z_lm_K_Cyt
Z_Shift_L_Gem
Z_lm_L_Gem
Z_Shift_L_Cyt
Z_lm_L_Cyt

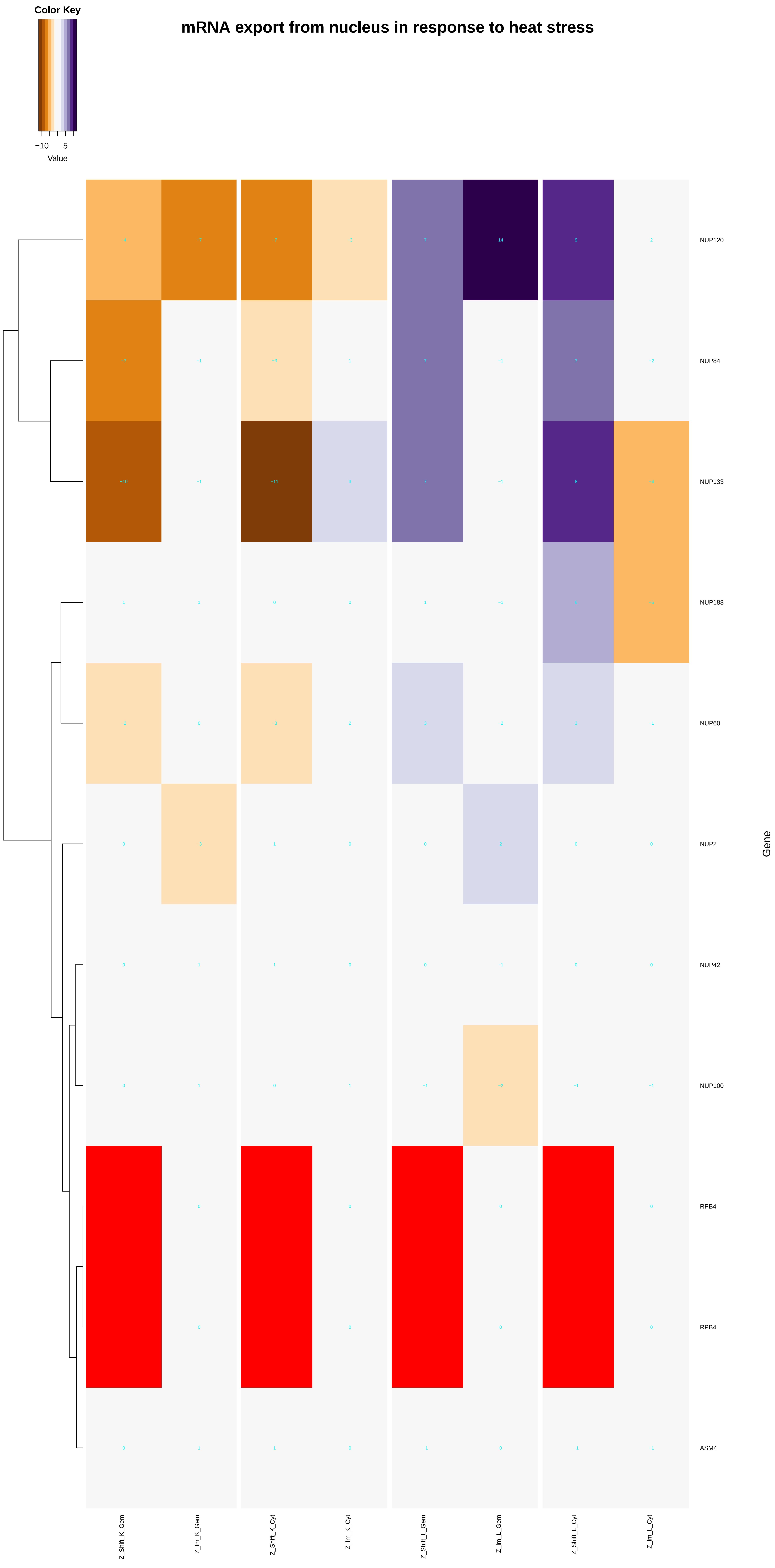
mRNA export from nucleus in response to heat stress
Color Key
−10
5
Value
NUP120
NUP84
NUP133
NUP188
NUP60
NUP2
NUP42
NUP100
RPB4
RPB4
ASM4
Gene
Z_Shift_K_Gem
Z_Im_K_Gem
Z_Shift_K_Cyt
Z_Im_K_Cyt
Z_Shift_L_Gem
Z_Im_L_Gem
Z_Shift_L_Cyt
Z_Im_L_Cyt

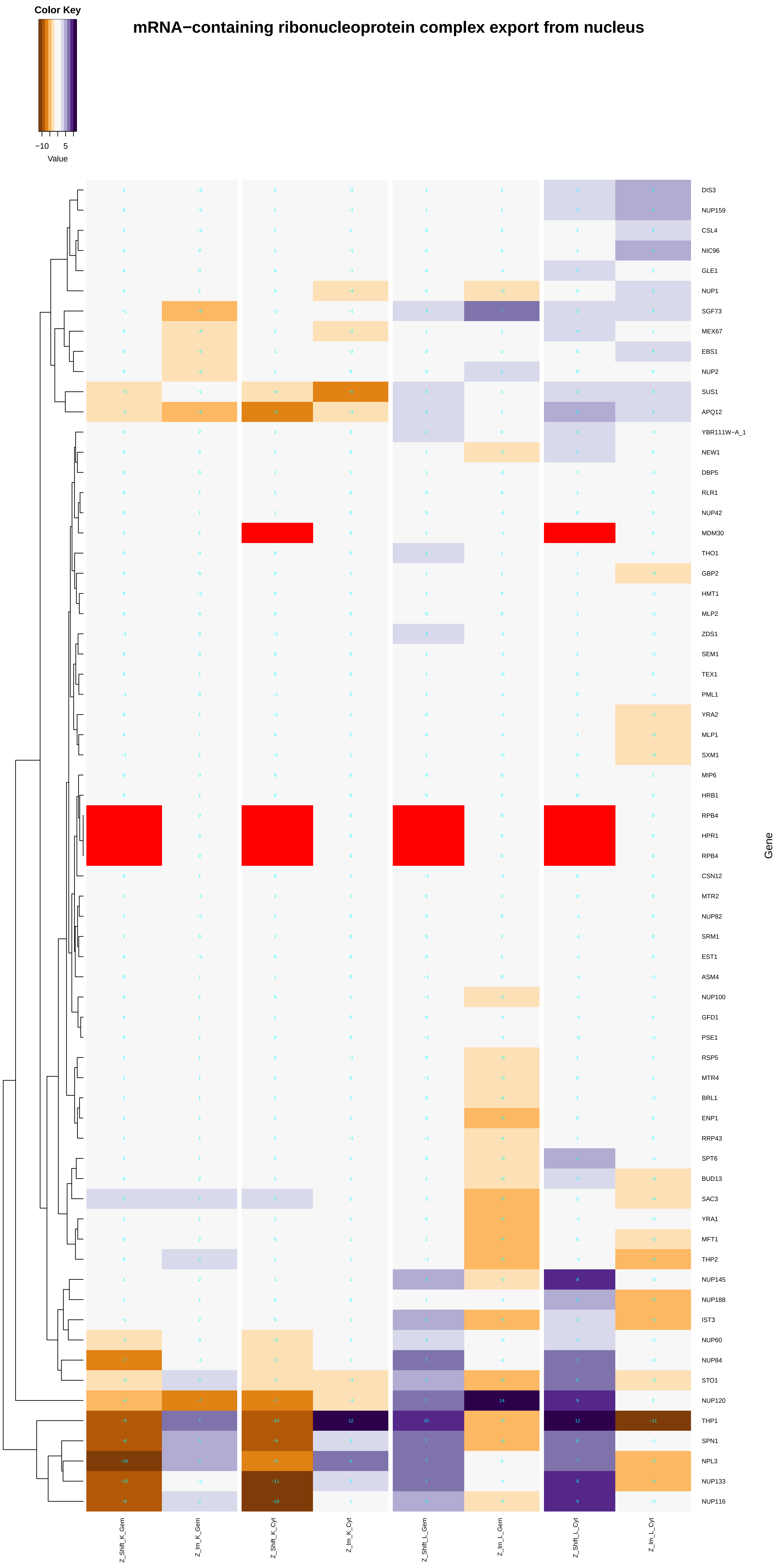
Color Key
−10
5
Value
mRNA−containing ribonucleoprotein complex export from nucleus
DIS3
NUP159
CSL4
NIC96
GLE1
NUP1
SGF73
MEX67
EBS1
NUP2
SUS1
APQ12
YBR111W−A_1
NEW1
DBP5
RLR1
NUP42
MDM30
THO1
GBP2
HMT1
MLP2
ZDS1
SEM1
TEX1
PML1
YRA2
MLP1
SXM1
MIP6
HRB1
RPB4
HPR1
RPB4
CSN12
MTR2
NUP82
SRM1
EST1
ASM4
NUP100
GFD1
PSE1
RSP5
MTR4
BRL1
ENP1
RRP43
SPT6
BUD13
SAC3
YRA1
MFT1
THP2
NUP145
NUP188
IST3
NUP60
NUP84
STO1
NUP120
THP1
SPN1
NPL3
NUP133
NUP116
Gene
Z_Shift_K_Gem
Z_Im_K_Gem
Z_Shift_K_Cyt
Z_Im_K_Cyt
Z_Shift_L_Gem
Z_Im_L_Gem
Z_Shift_L_Cyt
Z_Im_L_Cyt

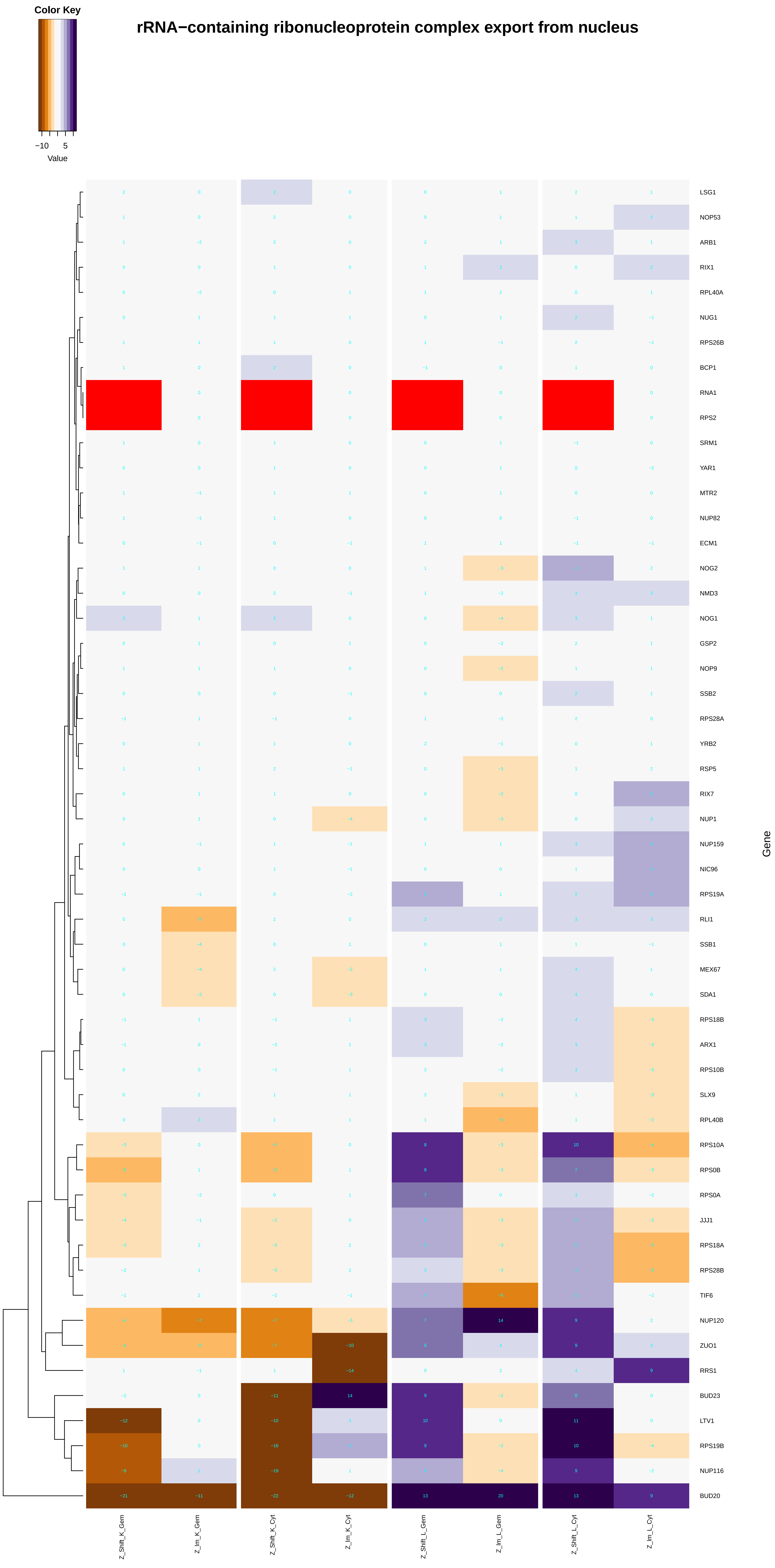

Color Key
−10
5
Value
rRNA−containing ribonucleoprotein complex export from nucleus
LSG1
NOP53
ARB1
RIX1
RPL40A
NUG1
RPS26B
BCP1
RNA1
RPS2
SRM1
YAR1
MTR2
NUP82
ECM1
NOG2
NMD3
NOG1
GSP2
NOP9
SSB2
RPS28A
YRB2
RSP5
RIX7
NUP1
NUP159
NIC96
RPS19A
RLI1
SSB1
MEX67
SDA1
RPS18B
ARX1
RPS10B
SLX9
RPL40B
RPS10A
RPS0B
RPS0A
JJJ1
RPS18A
RPS28B
TIF6
NUP120
ZUO1
RRS1
BUD23
LTV1
RPS19B
NUP116
BUD20
Gene
Z_Shift_K_Gem
Z_Im_K_Gem
Z_Shift_K_Cyt
Z_Im_K_Cyt
Z_Shift_L_Gem
Z_Im_L_Gem
Z_Shift_L_Cyt
Z_Im_L_Cyt

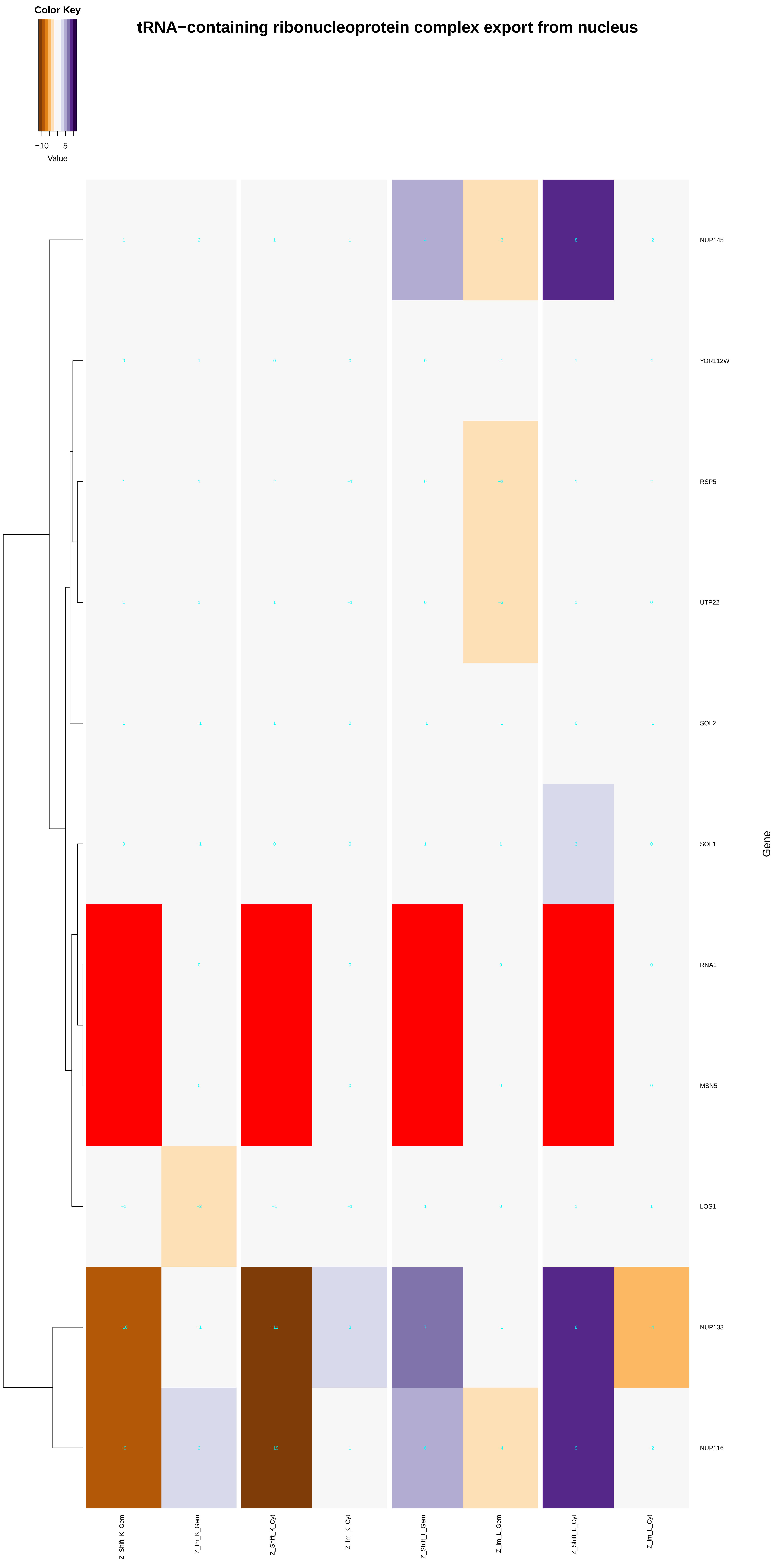
Color Key
−10
5
Value
tRNA−containing ribonucleoprotein complex export from nucleus
NUP145
YOR112W
RSP5
UTP22
SOL2
SOL1
RNA1
MSN5
LOS1
NUP133
NUP116
Gene
Z_Shift_K_Gem
Z_Im_K_Gem
Z_Shift_K_Cyt
Z_Im_K_Cyt
Z_Shift_L_Gem
Z_Im_L_Gem
Z_Shift_L_Cyt
Z_Im_L_Cyt

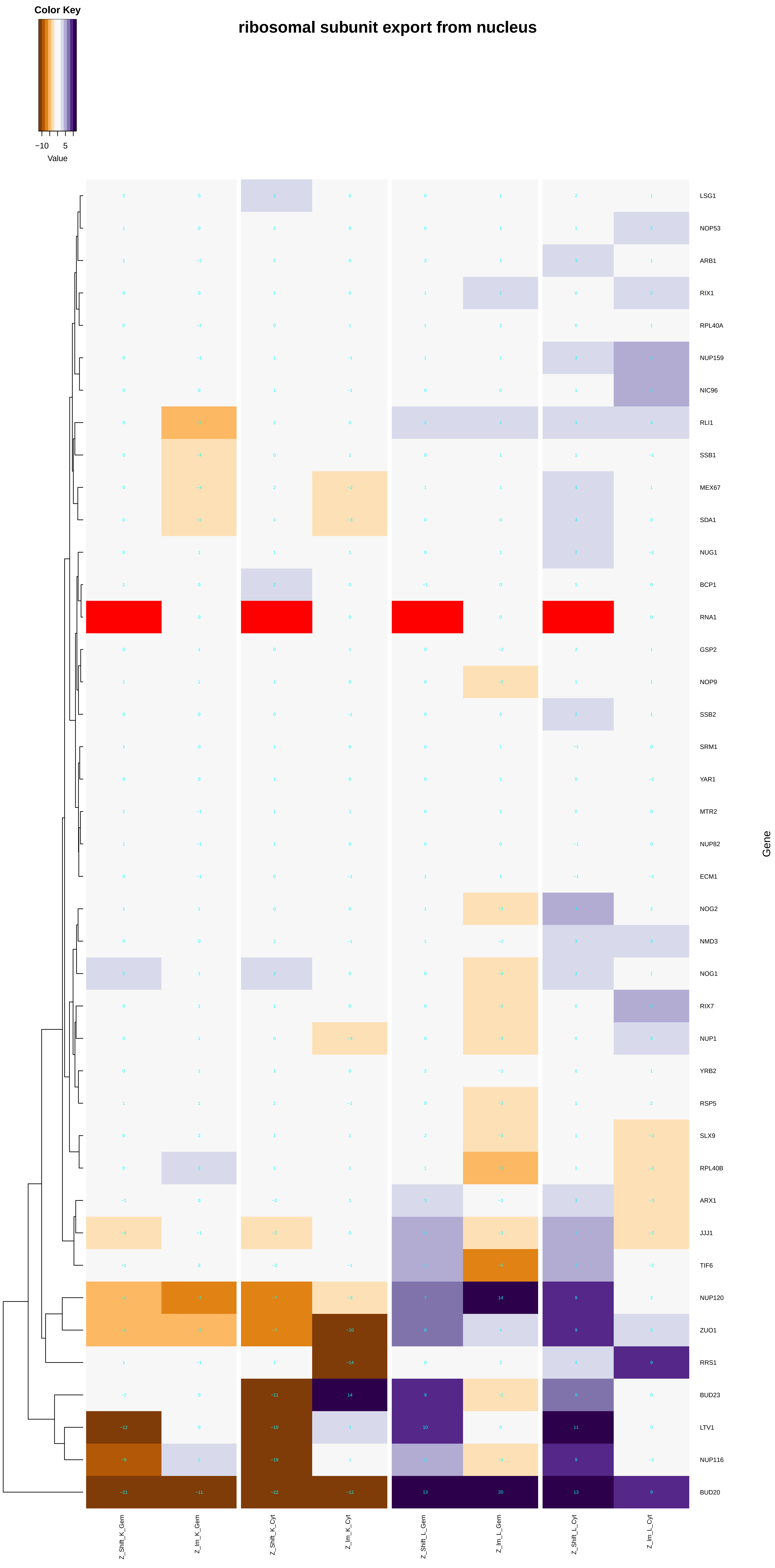

ribosomal subunit export from nucleus
Color Key
−10
5
Value
LSG1
NOP53
ARB1
RIX1
RPL40A
NUP159
NIC96
RLI1
SSB1
MEX67
SDA1
NUG1
BCP1
RNA1
GSP2
NOP9
SSB2
SRM1
YAR1
MTR2
NUP82
ECM1
NOG2
NMD3
NOG1
RIX7
NUP1
YRB2
RSP5
SLX9
RPL40B
ARX1
JJJ1
TIF6
NUP120
ZUO1
RRS1
BUD23
LTV1
NUP116
BUD20
Gene
Z_Shift_K_Gem
Z_lm_K_Gem
Z_Shift_K_Cyt
Z_lm_K_Cyt
Z_Shift_L_Gem
Z_lm_L_Gem
Z_Shift_L_Cyt
Z_lm_L_Cyt

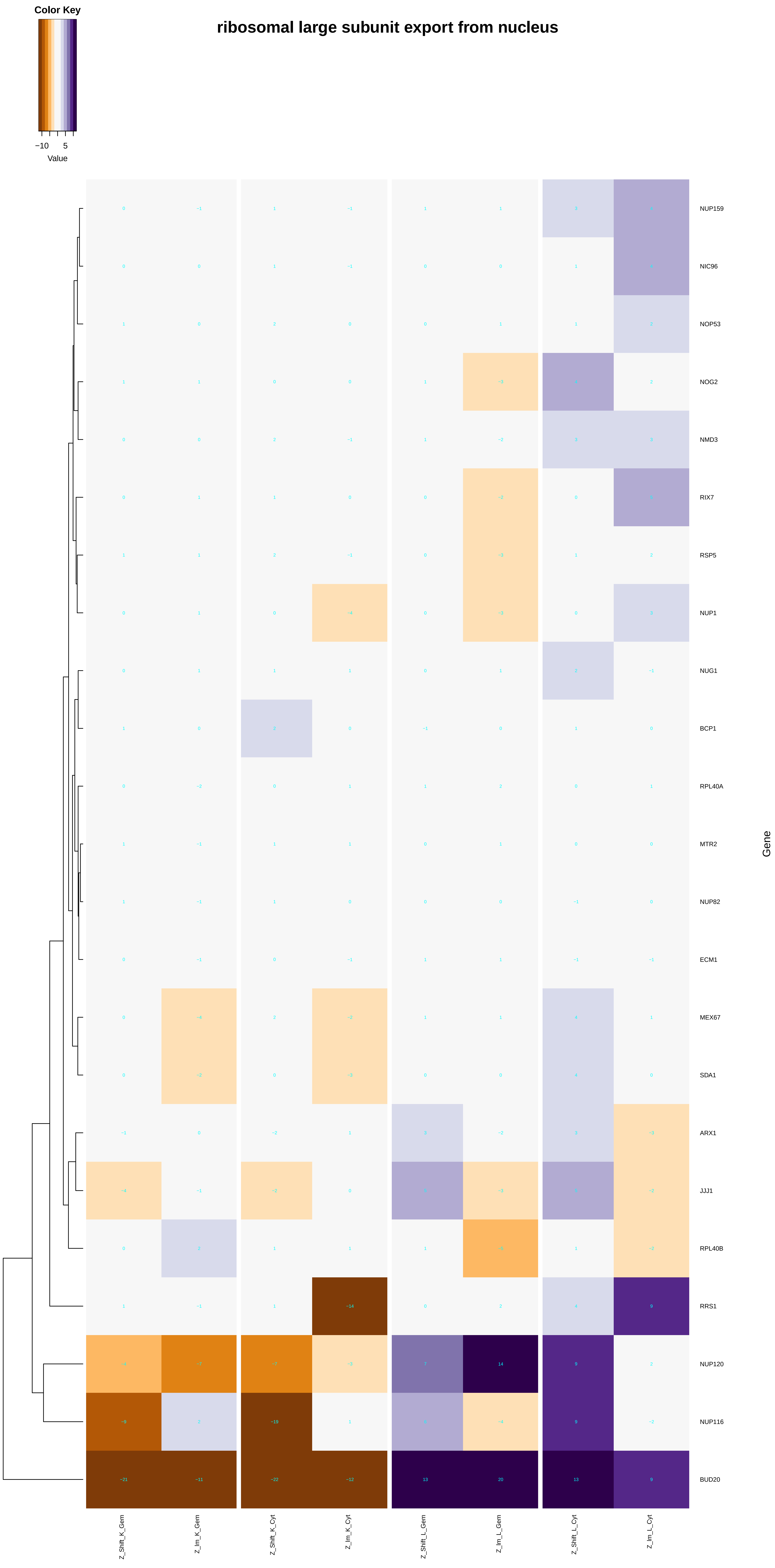

Color Key
−10
5
Value
ribosomal large subunit export from nucleus
NUP159
NIC96
NOP53
NOG2
NMD3
RIX7
RSP5
NUP1
NUG1
BCP1
RPL40A
MTR2
NUP82
ECM1
MEX67
SDA1
ARX1
JJJ1
RPL40B
RRS1
NUP120
NUP116
BUD20
Gene
Z_Shift_K_Gem
Z_Im_K_Gem
Z_Shift_K_Cyt
Z_Im_K_Cyt
Z_Shift_L_Gem
Z_Im_L_Gem
Z_Shift_L_Cyt
Z_Im_L_Cyt

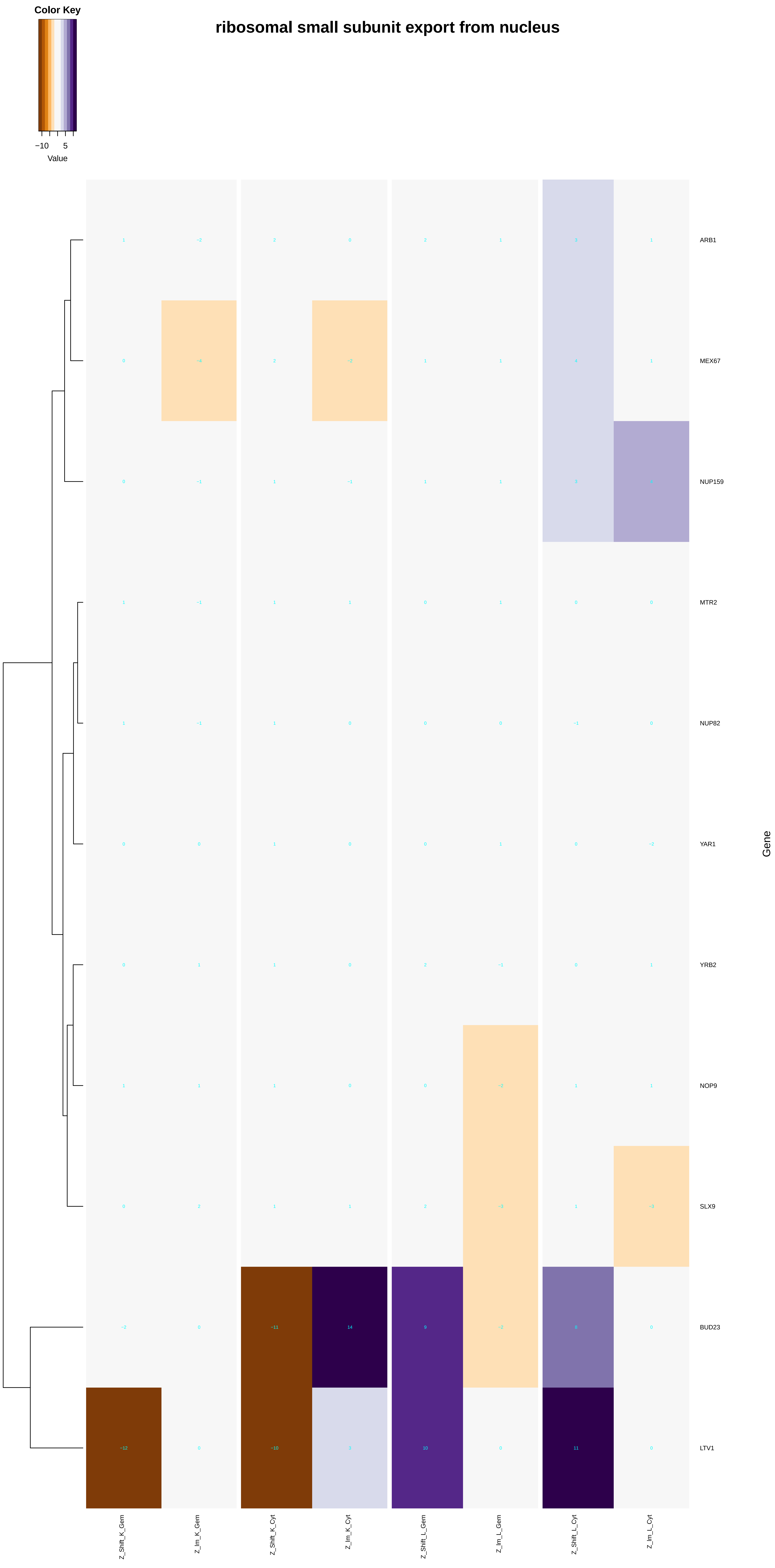
Color Key
−10
5
Value
ribosomal small subunit export from nucleus
ARB1
MEX67
NUP159
MTR2
NUP82
YAR1
YRB2
NOP9
SLX9
BUD23
LTV1
Gene
Z_Shift_K_Gem
Z_Im_K_Gem
Z_Shift_K_Cyt
Z_Im_K_Cyt
Z_Shift_L_Gem
Z_Im_L_Gem
Z_Shift_L_Cyt
Z_Im_L_Cyt
